# A computational model of the hypothalamic - pituitary - gonadal axis in female fathead minnows (*Pimephales promelas*) exposed to 17α-ethynylestradiol and 17β-trenbolone

**DOI:** 10.1186/1752-0509-5-63

**Published:** 2011-05-05

**Authors:** Zhenhong Li, Kevin J Kroll, Kathleen M Jensen, Daniel L Villeneuve, Gerald T Ankley, Jayne V Brian, María S Sepúlveda, Edward F Orlando, James M Lazorchak, Mitchell Kostich, Brandon Armstrong, Nancy D Denslow, Karen H Watanabe

**Affiliations:** 1 Division of Environmental and Biomolecular Systems, Oregon Health & Science University, Beaverton, OR, 97006, USA; 2 Department of Physiological Sciences and Center for Environmental and Human Toxicology, University of Florida, Gainesville, FL, 32611, USA; 3 U.S. EPA, Mid-Continent Ecology Division, Duluth, MN, 55804, USA; 4 Institute for the Environment, Brunel University, Uxbridge, Middlesex, UB8 3PH, UK; 5 Department of Forestry and Natural Resources, Purdue University, Lafayette, IN, 47907, USA; 6 Department of Animal & Avian Sciences, University of Maryland, College Park, MD 20742 USA; 7 U.S. EPA, Molecular Indicator Research Branch, Cincinnati, OH, 45268, USA; 8 The McConnell Group c/o U.S. EPA NERL EERD, USA

## Abstract

**Background:**

Endocrine disrupting chemicals (e.g., estrogens, androgens and their mimics) are known to affect reproduction in fish. 17α-ethynylestradiol is a synthetic estrogen used in birth control pills. 17β-trenbolone is a relatively stable metabolite of trenbolone acetate, a synthetic androgen used as a growth promoter in livestock. Both 17α-ethynylestradiol and 17β-trenbolone have been found in the aquatic environment and affect fish reproduction. In this study, we developed a physiologically-based computational model for female fathead minnows (FHM, *Pimephales promelas*), a small fish species used in ecotoxicology, to simulate how estrogens (i.e., 17α-ethynylestradiol) or androgens (i.e., 17β-trenbolone) affect reproductive endpoints such as plasma concentrations of steroid hormones (e.g., 17β-estradiol and testosterone) and vitellogenin (a precursor to egg yolk proteins).

**Results:**

Using Markov Chain Monte Carlo simulations, the model was calibrated with data from unexposed, 17α-ethynylestradiol-exposed, and 17β-trenbolone-exposed FHMs. Four Markov chains were simulated, and the chains for each calibrated model parameter (26 in total) converged within 20,000 iterations. With the converged parameter values, we evaluated the model's predictive ability by simulating a variety of independent experimental data. The model predictions agreed with the experimental data well.

**Conclusions:**

The physiologically-based computational model represents the hypothalamic-pituitary-gonadal axis in adult female FHM robustly. The model is useful to estimate how estrogens (e.g., 17α-ethynylestradiol) or androgens (e.g., 17β-trenbolone) affect plasma concentrations of 17β-estradiol, testosterone and vitellogenin, which are important determinants of fecundity in fish.

## Background

In vertebrates, such as fish, the hypothalamic-pituitary-gonadal (HPG) axis controls reproductive processes through a variety of hormones which act on target tissues directly or indirectly [1,2]. The HPG axis can be altered by endocrine disrupting chemicals (EDCs) in the aquatic environment which mimic endogenous hormones, alter their concentrations, or block their actions [3].

In recent years, many scientific studies have been conducted to study reproductive effects of EDCs in fathead minnow (FHM, *Pimephales promelas*), a model small fish species used in ecotoxicology [4-6]. Two EDCs, 17α-ethynylestradiol and 17β-trenbolone, have been widely studied as model estrogens and androgens, respectively [7-11]. Both compounds also are environmentally relevant contaminants.

17α-ethynylestradiol (EE_2_), a synthetic estrogen used in birth control pills, enters the environment mainly through effluents from wastewater treatment facilities. The reported median EE_2 _concentration in the aquatic environment varies from <0.5 to 15 ng/L [12]. Due in part to its high binding affinity for estrogen receptor (ER) [13-15], EE_2 _affects the HPG axis in FHM at environmentally relevant concentrations. Exposure to EE_2 _has been shown to result in altered hormone profiles, and increased vitellogenin (VTG, a precursor of egg yolk proteins) levels in both male and female FHMs [16]. In addition, a seven-year, whole-lake experiment conducted in Canada [17] showed that chronic exposure of FHMs to 5 - 6 ng EE_2_/L led to near-extinction of this species from the lake.

17β-trenbolone (TB) is a relatively stable metabolic product of trenbolone acetate, a synthetic androgen used as a growth promoter in livestock (e.g., cattle). TB enters the environment mainly as runoff from livestock feedlots. Schiffer et al. [18] reported that the TB concentration in effluents of solid cattle dung was around 19 ng/L. Durhan et al. [19] studied a cattle feedlot located in southwest central Ohio, and reported that the TB concentration in feedlot discharge was between 10 and 20 ng/L. TB has a high binding affinity for the androgen receptor (AR). Water exposure to TB at concentrations similar to those found in the environment decreases egg production in FHM in conjunction with changes in plasma concentrations of 17β-estradiol (E_2_), testosterone (T), and VTG in females [7]. Interestingly, relationships between TB water exposure concentrations and plasma E_2_, T and VTG concentrations were not monotonic, but were "U-shaped" [7].

To better understand the dynamics of the HPG axis in female FHMs and to facilitate the evaluation of adverse outcomes on reproduction from both estrogenic and androgenic EDC exposure, we developed a physiologically based computational model to simulate key reproductive endpoints, such as plasma concentrations of E_2_, T, and VTG, in adult female FHMs. The model simulates absorption, distribution, and elimination of TB and EE_2 _by incorporating salient physiological characteristics of FHMs and modelling biochemical pathways and reactions mathematically. This model is a first step toward predicting adverse outcomes on reproduction, which is an important component of ecological risk assessment. It robustly links TB and EE_2 _exposure to plasma steroid hormone and VTG concentrations, which can then be used to predict effects on fecundity. Though this model does not simulate oocyte growth dynamics to predict fecundity, it can be integrated with an oocyte growth dynamics model to do so. To our knowledge, it is the first physiologically based model capable of simulating exposure to a mixture of an estrogen and an androgen.

## Methods

### Model Formulation

We developed the HPG axis model for female FHM by modifying a computational model for male FHM described by Watanabe et al. [20]. The model simulates time continuously, but it does not have a seasonal component. In the following, we mainly focus on the unique formulations and/or assumptions in this model for female FHMs.

**Figure 1:**
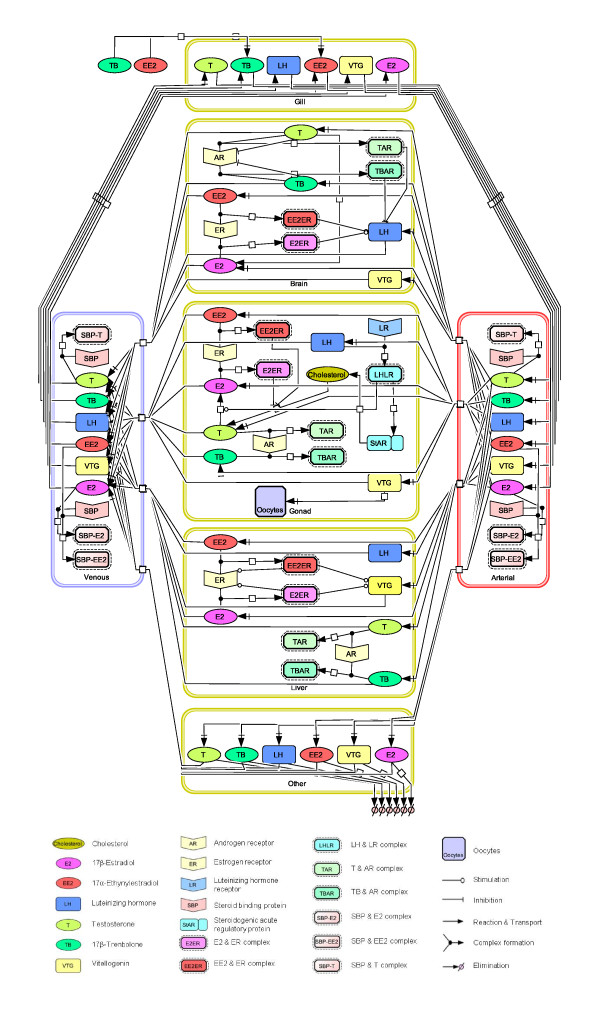
**Conceptual model of the HPG axis in adult female FHMs**. Tissues in adult female FHMs are categorized into six compartments: gill, brain, gonad, liver, venous blood, and other. Each compartment is defined by volume, blood flow, and partition coefficient, and performs multiple physiological functions.

The model for female FHMs contains six tissue compartments which represent organs or tissues important for absorption, distribution, metabolism, and elimination of exogenous and endogenous chemicals of interest (Figure 1). The six compartments are gill, brain, gonad, liver, venous blood and "other". In the arterial blood, the concentrations of both free and bound chemicals are equal to those in the venous blood compartment, unless a chemical(s) enters the body through a water exposure. As a result, we did not count arterial blood as an independent compartment. Based upon a mass balance for each chemical of interest, a set of coupled ordinary differential equations were formulated in each compartment following the principles of physiologically based pharmacokinetic modeling. A detailed description of the differential equations can be found in Additional File 1: Differential equations used in the HPG axis model.

In the brain, gonad, and liver compartments, we simulated both ER and AR dynamics. The AR component was not included in the model for male FHM published by Watanabe et al. [20]. ER binds estrogens (e.g., E_2 _and EE_2_), and bound ER affects the production of VTG. AR binds androgens (e.g., T and TB), and subsequently regulates biochemical processes such as the production of gonadotropins [21]. A general mathematical formulation of ligand-receptor binding is shown in Equation 1.(1)

where, *C*_iR, *j *_(nmol/L) is the concentration of compound *i *(e.g. T, TB, E_2 _and EE_2_) bound to its receptor in compartment *j *(e.g. brain, liver, gonad, and venous blood) ; *V_j _*(L) is the volume of compartment *j*; *k*_1_iR, *j *_(L/nmol/hr) is the association rate constant of compound *i *with its receptor in compartment *j*; *C_i, j _*(nmol/L) is the concentration of free compound *i *in compartment *j*; *C_R, j _*(nmol/L) is the concentration of unbound receptor of compound *i *in compartment *j*; *K*_d_iR, *j *_(nmol/L) is the equilibrium dissociation constant of compound *i *with its receptor in compartment *j*.

#### Gill

In the gill compartment, we did not simulate any production of proteins (e.g., VTG), hormones (e.g., luteinizing hormone, LH), or hormone receptors (e.g, ER and AR). ER mRNA is present in FHM gills, however, we did not simulate ER in the gill compartment because the gill expression of ER is very low compared to other tissues [22]. We simulated the exposure of female FHMs to TB and/or EE_2 _in water, and the gill compartment is where the exogenous chemicals are absorbed. The concentration of each chemical in exposure water was represented as a function of time. Then, equilibrium partitioning was assumed, and the FHM arterial blood concentration was calculated from the water concentration using an equilibrium partition coefficient assigned for each chemical (Equation 2). In addition, we assumed that the gill compartment did not accumulate any chemical(s).(2)

where, CArt*_i _*(nmol/L) is the concentration of exogenous chemical *i *in arterial blood; FW_gil _(L/hr) is the volumetric flow rate of water through the gills; *C*_*i*, H20 _(nmol/L) is the concentration of exogenous chemical *i *(e.g. TB and EE_2_) in exposure water as a function of time; *F*_car _(L/hr) is cardiac output; and CVen*_i _*(nmol/L) is the concentration of exogenous chemical *i *in venous blood; λ_*i*, bld _is the partition coefficient for exogenous chemical *i *between blood and water. Partition coefficients are often determined experimentally, but when a measured or model-estimated value is unavailable, we calibrate it to fit the experimental data.

#### Brain

In the brain compartment, three key assumptions were made: (i) the down-regulation of LH (gonadotropin II) synthesis by bound AR [23,24]; (ii) the up-regulation of LH synthesis by bound ER [25]; and (iii) the down-regulation of AR synthesis by free androgens [26,27].

In the brain, androgens have a negative feedback on the synthesis and release of gonadotropin releasing hormone (GnRH) [23], which in turn controls the synthesis of gonadotropins. To investigate how androgens may regulate GnRH, we searched for an androgen response element (ARE) in the promoter regions of *gnrh *genes. Due to a lack of information on gene promoter sequences in FHM, we conducted the search in zebrafish (*Danio rerio*), a cyprinid fish closely related to FHM. We found that *gnrh *promoters contain several ARE half sites (tgttct) [24]. Thus, we postulated that androgens have a negative control on GnRH synthesis mainly through bound AR. However, we did not have any measurements of GnRH in FHM and GnRH was not included in the model, so we formulated a down-regulation of LH synthesis by bound AR in the model.

Second, we assumed an up-regulation of LH synthesis by bound ER in the brain compartment. This assumption was based upon observations of estrogen response elements (EREs) in the promoter region of the *lh *gene and reports of estrogen-stimulated LH production in fish [25]. Equation 3 describes the LH production rate in the brain compartment as a function of bound AR and ER. In the equation, *P*_LH, brn _(nmol/hr) is the production rate of LH in brain; *P*_b_LH, brn _(nmol/hr) is the background production rate of LH in brain, which was formulated as a diurnal cycle; *C*_ER_bd, brn _(nmol/L) is the total concentration of bound ER in brain, which equals the sum of E_2_- and EE_2_- bound ER concentrations; *ρ*_u_LH, brn _(nmol/L) is an induction factor for LH production by bound ER; *C*_AR_bd, brn _(nmol/L) is the total concentration of bound AR in brain, which equals the sum of T- and TB- bound AR concentrations ; *ρ*_d_LH, brn _(nmol/L) is a factor for inhibition of LH production by bound AR.(3)

The brain compartment is also very important for the regulation of AR production [26,27]. In mammals (e.g. rats, mice, and human), AR mRNA in brain is down-regulated by androgens, such as T and dihydrotestosterone [26,27], though little is known about the corresponding mechanisms. We searched for AREs in the promoter region of the *ar *gene in zebrafish, but did not find any match. Hence, we postulated that the down-regulation of AR mRNA by androgens is associated with a non-genomic pathway [28], or associated with cell factors other than the soluble AR simulated in our model [29]. Thus, we assumed a down-regulation of AR production by free androgens in the brain compartment. When ARs are produced, some bind androgens, some remain unbound, and others degrade. Based upon a mass balance for free AR, Equation 4 describes the processes of AR production, association and dissociation with T or TB, and degradation.(4)

where, *C*_AR_free, brn _(nmol/L) is the free AR concentration in brain; Pbg_AR, brn _(nmol/L/hr) is the background production rate of AR in brain; *C*_*T*, brn _(nmol/L) is the free T concentration in brain; *C*_TB, brn _(nmol/L) is the free TB concentration in brain; *K*_AR, brn _(nmol/L) is an inhibition rate constant for AR production by free T and TB; *k*_1_TAR, brn _(L/nmol/hr) is the association rate constant for T and AR; K_d_TAR, brn _(nmol/L) is the dissociation rate constant for T bound to AR; *C*_TAR, brn _(nmol/L) is the concentration of the T-AR complex in brain; *k*_1_TBAR, brn _(L/nmol/hr) is the association rate of TB to AR; K_d_TBAR, brn _(nmol/L) is the dissociation rate constant for TB bound to AR; *C*_TBAR, brn _(nmol/L) is the concentration of the TB-AR complex in brain; and *k*_e___AR, brn _(1/hr) is the elimination rate for free AR. We included the inhibition of LH by bound AR and the inhibition of AR by free androgens to account for the U-shaped dose-response curves for plasma E_2_, T and VTG concentrations observed in female FHMs exposed to TB [7]. These assumptions and mathematical formulations provided a robust fit to the available data [7]. An alternate formulation based upon brain AR and gonad AR with different binding affinities was tried first. However, because of a lack of parameter information and evidence for the biological mechanism, we abandoned this approach for the present version. The present model formulation makes more sense biologically, and is simpler.

#### Gonad

In the gonad compartment, modifications to the model formulations for male FHMs include (i) absorption of VTG into oocytes; and (ii) up-regulation of E_2 _production by bound LH. The absorption of VTG into oocytes was formulated as a first order kinetic process. VTG is synthesized in the liver [30], and circulates to the gonads where it is taken up via receptor-mediated endocytosis into oocytes, and then processed into yolk proteins [31]. Although the molecular mechanism of VTG uptake is known, we did not have data to describe this process quantitatively. As a result, a first order kinetic equation with an assumed first order rate constant was formulated to represent the process (Equation 5).(5)

where, *R*_VTG, gon _(nmol/hr) is the absorption rate of VTG into oocytes in the gonad compartment; *k*_VTG, gon _(1/hr) is the absorption rate constant for VTG into oocytes in the gonad compartment; *C*_VTG, gon _(nmol/L) is the concentration of VTG in the gonad compartment; and *V*_gon _(1/L) is the volume of the gonad compartment.

Secondly, we simulated an up-regulation of E_2 _production by bound LH in the gonad compartment. It was observed that LH stimulates the activity and gene expression of aromatase in the gonads of teleosts [32]. In our model, we formulated the regulation of E_2 _production as being proportional to the concentration of bound LH in the gonad compartment (Equation 6).(6)

where, *P*_E2, gon _(nmol/hr) is the rate of E_2 _production; *ρ*_E2_LHLR, gon _(L/nmol) is an induction factor of E_2 _production by bound LH; *C*_LHLR, gon _(nmol/L) is the concentration of bound LH; Vmax_aro, gon _(nmol/hr) is the maximum rate of E_2 _production by gonad aromatase; Km_aro, gon _(nmol/L) is the Michaelis-Menten constant for gonad aromatase; *C*_*T*, gon _(nmol/L) is the concentration of T.

#### Liver

In the liver compartment, formulations including ER auto-regulation and bound-ER-stimulated VTG production are the same as those described by Watanabe et al. [20], except that we added ligand-receptor binding of T and TB to the AR.

#### Venous blood

Besides E_2 _and T, we simulated the association and dissociation processes of EE_2 _to steroid-binding proteins (SBPs) in the venous blood compartment. There is contradictory information about the binding affinities of EE_2 _to SBPs in fish. Compared to E_2_, some fish species such as channel catfish (*Ictalurus punctatus*) and zebrafish (*Danio rerio*) have high binding affinity of EE_2 _to SBPs [33,34], while other fish species such as Arctic charr (*Salvelinus alpinus*) have a low binding affinity [35]. To date, binding affinity measurements of EE_2 _to SBPs in FHM have not been made. Watanabe et al. [20] did not include the binding process of EE_2 _to SBPs in blood. In their modelling work for male FHMs, the total concentration of SBPs was assumed to be 20 nmol/L based upon a measurement in human males [36]. Such a low value has little effect on free plasma EE_2 _concentration or model performance. However, in our model for female FHMs, we assumed the total concentration of SBPs to be 400 nmol/L [36,37] based upon SBP measurements in female spotted seatrout (*Cynoscion nebulosus*) [37] and in human females [36]. Consequently, a large amount of EE_2 _could be bound by SBPs in blood, which would affect the total concentration of EE_2 _in the plasma. Therefore, we included the binding process of EE_2 _to SBPs in this model, and formulated it using Equation 1.

#### Other

In our model, the 'Other' compartment is where elimination of exogenous and endogenous chemicals and proteins occur. Besides E_2_, EE_2_, T, VTG, and LH (included in Watanabe et al. [20]), we added a first order kinetic equation to describe the elimination of TB, and the first order elimination rate constant was assumed to be the same as that of EE_2 _(Equation 7).(7)

where *R*_TB, oth _(nmol/hr) is the elimination rate of TB in the Other compartment; *k*_e___TB, oth _(1/hr) is the elimination rate constant for TB in the Other compartment; *C*_TB, oth _(nmol/L) is the concentration of TB in the Other compartment; and *V*_oth _(1/L) is the volume of the Other compartment.

### Experimental Data

To calibrate model parameters and to evaluate model predictions, we used data from unexposed, TB-exposed, and EE_2_-exposed adult female FHMs from 18 different studies. All studies were conducted with sexually mature (five to seven month old) female FHMs. Chemical exposures were conducted in the laboratory under optimal conditions for FHM reproduction. For example, the temperature was 25°C, photoperiod was 16/8 hr (light to dark), and food was not limited. Under such conditions, FHMs can remain in reproductive condition and spawn year around. For each fish, physiological parameters, including body weight (BW), gonadosomatic index (GSI), and hepatosomatic index (HSI), were input into the model. For all experimental data used in model calibration or validation, when any measurements of BW, GSI, or HSI were missing, we used the medians of measured BW, GSI, or HSI, respectively [38]. Ideally, if all experimental data had been available when we started to develop the model in 2006, we would have randomly selected a subset of data from each experiment for model calibration and used the remainder for model evaluation. However, several of the experimental studies were conducted while the model was being developed. Thus, we used data as they became available. The following summarizes the experimental data and how the data were used.

The reproductive endpoint data in unexposed (control) adult female FHMs were obtained from an earlier paper by Watanabe et al. [38]. In a total of 170 female FHMs, the data include measurements of plasma E_2_, T, and VTG concentrations; all measurements were made in some fish, and in others only a subset of endpoints (e.g., plasma E_2 _and VTG concentrations) were measured. We randomly split the data; the first 75 records were used to calibrate our model; the remaining 95 records were used in model validation.

Experimental data from TB-exposed adult female FHMs were obtained from three studies: (i) a flow-through water exposure to nominal concentrations of 0.005, 0.05, 0.5, 5.0, and 50 μg TB/L for 21 days by Ankley et al. [7]; (ii) a static exposure to nominal concentrations of 0.05, 0.5, 5 μg TB/L for 48 hours by Garcia-Reyero et al. [39]; and (iii) a flow-through water exposure to nominal concentrations of 0.05 and 0.5 μg TB/L in adult female FHMs for eight days, followed by an eight-day depuration described by Ekman et al. [40]. In Ankley et al. [7], 12 female FHMs were exposed in each treatment group. On the 21^st ^day of exposure, all FHMs were sacrificed; plasma concentrations of E_2_, T, and VTG were measured in each fish. In Garcia-Reyero et al. [39] eight FHMs were exposed in each treatment group. After a 48-hour exposure, the fish were sacrificed. For each treatment group, plasma E_2 _concentrations were measured in each of four fish, and plasma VTG concentrations were measured in each of the four remaining fish. The concentrations of VTG and E_2 _were not measured in the same fish because Dr. Orlando's laboratory measured E_2 _and Dr. Denslow's laboratory measured VTG. In Ekman et al. [40], 64 FHMs were exposed to TB in each treatment group. On the 1^st^, 2^nd^, 4^th^, and 8^th ^day of exposure and the 1^st^, 2^nd^, 4^th^, and 8^th ^day of depuration (test days 9, 10, 12, and 16), for each treatment group, eight FHMs were sacrificed to measure plasma E_2 _and VTG concentrations in each fish. Data from Ankley et al. [7] were used to calibrate our model, and data from Garcia-Reyero et al. [39] and Ekman et al. [40] were used to evaluate our model predictions.

VTG plasma concentrations in adult female FHMs exposed to EE_2 _were obtained from three studies: (i) a flow-through water exposure to nominal concentrations of 10 or 100 ng EE_2_/L in adult female FHMs for eight days, followed by an eight-day depuration [41]; (ii) a flow-through water exposure to a nominal concentration of 0.5, 1.5, and 4.5 ng EE_2_/L in adult female FHMs for 21 days by Lazorchak et al. [42]; and (iii) a flow-through water exposure to a nominal concentration of 1.5 ng EE_2_/L in adult female FHMs for 21 days by Brian et al. [43]. In Ekman et al. [41], for each treatment group and each sampling time, eight FHMs were sacrificed to measure plasma VTG concentration in each fish. Sampling occurred on the 1^st^, 4^th^, and 8^th ^day of exposure to EE_2_, and the 8^th ^day of EE_2 _depuration (test day 16). In Lazorchak et al. [42], 28 FHMs in each of the treatment groups (0.5, 1.5, and 4.5 ng EE_2_/L) were sacrificed to measure plasma VTG concentration in each fish on the 21^st ^day. In Brian et al. [43], four FHMs were sacrificed to measure plasma VTG concentration in each fish on the 21^st ^day after exposure to 1.5 ng EE_2_/L. As opposed to the three TB and two EE_2 _studies which did not use carrier solvents, Brian et al. [39] used N, N-dimethylformamide, DMF, as a chemical carrier for EE_2_. Data from Ekman et al. [41] were used to calibrate our model, and data from Lazorchak et al. [42], and Brian et al. [43] were used to evaluate our model predictions.

### Model Calibration

In total, our model contains 123 input parameters, such as volume and blood flow rates of each compartment, chemical equilibrium partition coefficients, ligand-receptor association and dissociation rate constants, and kinetic rate constants for each biochemical reaction. The parameters were fixed with known values, or calibrated using experimental data collected in adult female FHMs. In total, 97 model parameters were fixed with values obtained from published literature or measured for this study (Table 1). The remaining 26 model parameters were calibrated using Markov Chain Monte Carlo simulation [44-47], which requires the definition of prior distributions for each parameter being calibrated.

**Table 1:** Model parameters treated as constants (*n *= 97)

Parameter description	Symbols	Value	Reference
Body weight^a^	BodyWt	0.0016 (kg)	Watanabe et al. [38]

Volumetric water flowing through gills	FW_gil_	10.6× BodyWt ^0.75 ^(L/hr)	Nichols et al. [57]

Cardiac output	F_car_	2.06× BodyWt ^0.75 ^(L/hr)	Nichols et al. [57]

Percentage of brain to body weight (BSI)	P_brn_	1.18	Measured by D. Villeneuve

Percentage of gonads to body weight (GSI)^b^	P_gon_	11	Watanabe et al. [38]

Percentage of liver to body weight (HSI)^c^	P_liv_	3.0	Watanabe et al. [38]

Percentage of gills to body weight	P_gil_	1.67	Nichols et al. [58]

Percentage of venous blood to body weight	P_ven_	2.59	Robinson et al. [59] Nichols et al. [58]

Percentage of "other" to body weight	P_oth_	= 100- P_brn_- P_gon_- P_liv_- P_gil_- P_ven_	Watanabe et al. [20]

Fraction of blood flow in brain to cardiac output			Nichols et al. [58]

Fraction of blood flow in gonad to cardiac output			Nichols et al. [58]

Fraction of blood flow in liver to cardiac output			Nichols et al. [58]

Fraction of blood flow in "other" to cardiac output			Nichols et al. [58]

Fraction of plasma in venous blood	F_plasma, ven_	0.45	Measured by K. Kroll

Total concentration of estrogen receptors in brain	C_ER, brn_	14.3 (nmol/L tissue)	Plowchalk and Teaguarden [60]

Total concentration of estrogen receptors in gonad	C_ER, gon_	29 (nmol/L tissue)	Plowchalk and Teaguarden [60]

Total concentration of LH receptors in gonad	C_LR, gon_	2.0 (nmol/L tissue)	Miwa et al. [61]

Total concentration of SBP in blood	C_SBP, ven_	400 (nmol/L blood)	Laidley and Thomas[37] Teeguarden and Barton [36]

Total concentration of AR in gonad	C_AR, gon_	1.05 (nmol/L tissue)	Sperry and Thomas [62]

Total concentration of AR in liver	C_AR, liv_	= C_AR, gon_	assumed

Association rate of E_2 _to estrogen receptor in brain	*k*_1_E2ER, brn_	0.743	Murphy et al. [63]

Dissociation constant of E_2 _to estrogen receptor in gonad	*K*_d_E2ER, gon_	= *K*_*d*_E2ER, brn_	assumed

Association rate of E_2 _to estrogen receptor in gonad	*k*_1_E2ER, gon_	= *k*_*1*_E2ER, brn_	assumed

Dissociation constant of E_2 _to estrogen receptor in liver	*K*_d_E2ER, liv_	= *K*_*d*_E2ER, brn_	assumed

Association rate of E_2 _to estrogen receptor in liver	*K*_1_E2ER, liv_	= *k*_*1*_E2ER, brn_	assumed

Dissociation constant of EE_2 _to estrogen receptor in brain	*K*_d_EE2ER, brn_	= *K*_*d*_E2ER, brn_/RBA_EE2_E2_	Denny et al. [15]

Association rate of EE_2 _to estrogen receptor in brain	*k*_1_EE2ER, brn_	= *k*_*1*_E2ER, brn_	assumed

Dissociation constant of EE_2 _to estrogen receptor in gonad	*K*_d_EE2ER, gon_	= *K*_*d*_EE2ER, brn_	assumed

Association rate of EE_2 _to estrogen receptor in gonad	*k*_1_EE2ER, gon_	= *k*_*1*_EE2ER, brn_	assumed

Dissociation constant of EE_2 _to estrogen receptor in liver	*K*_d_EE2ER, liv_	= *K*_*d*_EE2ER, brn_	assumed

Association rate of EE_2 _to estrogen receptor in liver	*k*_1_EE2ER, liv_	= *k*_*1*_EE2ER, brn_	assumed

Dissociation constant of T to androgen receptor in brain	*K*_d_TAR, brn_	3 (nmol/L)	Sperry and Thomas [62]

Association rate of T to androgen receptor in brain	*k*_1_TAR, brn_	0.08 (L/nmol/hr)	Sperry and Thomas [62]

Dissociation constant of T to androgen receptor in gonad	*K*_d_TAR, gon_	= *K*_*d*_TAR, brn_	assumed

Association rate of T to androgen receptor in gonad	*k*_1_TAR, gon_	= *k*_*1*_TAR, brn_	assumed

Dissociation constant of T to androgen receptor in liver	*K*_d_TAR, liv_	= *K*_*d*_TAR, brn_	assumed

Association rate of T to androgen receptor in liver	*k*_1_TAR, liv_	= *k*_*1*_TAR, brn_	assumed

Dissociation constant of TB to androgen receptor in brain	*K*_d_TBAR, brn_	= *K*_*d*_TAR, brn_/RBA*_TB_T_*	Wilson et al. [64]

Association rate of TB to androgen receptor in brain	*k*_1_TBAR, brn_	= *k*_*1*_TAR, brn_	assumed

Dissociation constant of TB to androgen receptor in gonad	*K*_d_TBAR, gon_	= *K*_*d*_TBAR, brn_	assumed

Association rate of TB to androgen receptor in gonad	*k*_1_TBAR, gon_	= *k*_*1*_TBAR, brn_	assumed

Dissociation constant of TB to androgen receptor in liver	*K*_d_TBAR, liv_	= *K*_*d*_TBAR, brn_	assumed

Association rate of TB to androgen receptor in liver	*k*_1_TBAR, liv_	= *k*_*1*_TBAR, brn_	assumed

Dissociation constant of E_2 _to SBP in blood	*K*_d_E2SBP, ven_	3.13 (nmol/L)	Murphy et al. [63]

Association rate of E_2 _to SBP in blood	*k*_1_E2SBP, ven_	5.6687 (L/nmol/hr)	Murphy et al. [63]

Dissociation constant of T to SBP in blood	*K*_d_TSBP, ven_	4.89 (nmol/L)	Murphy et al. [63]

Association rate of T to SBP in blood	*K*_1_TSBP, ven_	5.6687 (L/nmol/hr)	Murphy et al. [63]

Dissociation constant of EE_2 _to SBP in blood	*K*_d_EE2SBP, ven_	0.58 (nmol/L)	Miguel-Queralt and Hammond [34]

Association rate of EE_2 _to SBP in blood	*k*_1_EE2SBP, ven_	5.6687 (L/nmol/hr)	Murphy et al. [63]

Dissociation constant of LH to LH receptor in gonad	*K*_d_LHLR, gon_	2.9 (nmol/L)	Crim et al. [65]

Association rate of LH to LH receptor in gonad	*k*_1_LHLR, gon_	0.2 (L/nmol/hr)	Watanabe et al. [20]

Scaling coefficient of Vmax of T production in gonad (= Vmax/bodyweight ^0.75^)	sc_Vmax_Scc, gon_	1.1e+05 (nmol/hr/kg body weight)	Kashiwagi et al. [66]; Shikita and Hall [67]

K_0.5 _of T production in gonad	K_0.5Scc, gon_	190 (nmol/L)	Shikita and Hall [67]

Inhibition constant of T production by bound ER	K_T_	0.016	Watanabe et al. [20]

Km of E_2 _production in gonad	Km_aro, gon_	9.6 (nmol/L)	Zhao et al. [68]

Concentration of microsomal protein in gonads	D_mp, gon_	3100 (mg/L)	Measured by D. Villeneuve

Ratio between the concentrations of microsoaml protein in gonads and brain	Rho_mp_	0.174	Measured by D. Villeneuve

Scaling coefficient of Vmax of E_2 _production in brain (= Vmax/mass of microsomal protein in brain)	sc_Vmax_aro, brn_	= 4.6× sc_Vmax_aro, gon_	Zhao et al. [68]

Km of E_2 _production in brain	Km_aro, brn_	9.6 (nmol/L)	Zhao et al. [68]

Concentration of microsomal protein in brain	D_mp, brn_	= D_mp, gon_/Rho_mp_	Measured by D. Villeneuve

Ratio between concentrations of STAR and bound LR in gonads	Rho_STAR, gon_	1	assumed

Rate constant for Vtg uptake into oocytes	*k*_vtg, gon_	0.05	assumed

K_0.5 _of Vtg production in liver production	K_0.5Vtg, liv_	1.0 (nmol/L)	Watanabe et al. [20]

Elimination rate constant for ER in the liver compartment	*k*_e___ER, liv_	0.01 (1/hr)	Murphy et al. [63]

Elimination rate constant for AR in the brain compartment	*k*_e___AR, brn_	0.01 (1/hr)	Assumed

Elimination rate constant for LH in the "other" compartment	*k*_e___LH, oth_	0.1 (1/hr)	Teeguarden and Barton [36]

Elimination rate constant for E_2_ in the "other" compartment	*k*_e___E2, oth_	0.1 (1/hr)	Teeguarden and Barton [36]

Elimination rate constant for T in the "other" compartment	*k*_e___T, oth_	0.1 (1/hr)	Teeguarden and Barton [36]

Elimination rate constant for EE_2_ in the "other" compartment	*k*_e___EE2, oth_	0.1 (1/hr)	Teeguarden and Barton [36]

Elimination rate constant for TB in the "other" compartment	*k*_e___TB, oth_	0.1 (1/hr)	Teeguarden and Barton [36]

Elimination rate constant for Vtg in the "other" compartment	*k*_e___Vtg, oth_	0.001 (1/hr)	Teeguarden and Barton [36]

Partition coefficient of LH (brain to blood)	*λ*_LH, brn_	1	Teeguarden and Barton [36]

Partition coefficient of LH (gonad to blood)	*λ*_LH, gon_	1	Teeguarden and Barton [36]

Partition coefficient of LH (liver to blood)	*λ*_LH, liv_	1	Teeguarden and Barton [36]

Partition coefficient of LH ("other" to blood)	*λ*_LH, oth_	1	Teeguarden and Barton [36]

Partition coefficient of VTG (brain to blood)	*λ*_VTG, brn_	1	Teeguarden and Barton [36]

Partition coefficient of VTG (gonad to blood)	*λ*_VTG, gon_	1	Teeguarden and Barton [36]

Partition coefficient of VTG (liver to blood)	*λ*_VTG, liv_	1	Teeguarden and Barton [36]

Partition coefficient of VTG ("other" to blood)	*λ*_VTG, oth_	1	Teeguarden and Barton [36]

Partition coefficient of EE_2_ (blood to water)	*λ*_EE2, bld_	300	Watanabe et al. [20]

Partition coefficient of EE_2_ (brain to blood)	*λ*_EE2, brn_	1	Teeguarden and Barton [36]

Partition coefficient of EE_2_ (gonad to blood)	*λ*_EE2, gon_	1	Teeguarden and Barton [36]

Partition coefficient of EE_2_ (liver to blood)	*λ*_EE2, liv_	3	Watanabe et al. [20]

Partition coefficient of EE_2_ ("other" to blood)	*λ*_EE2, oth_	1	Teeguarden and Barton [36]

Partition coefficient of E_2_ (blood to water)	*λ*_E2, bld_	300	Watanabe et al. [20]

Partition coefficient of E_2_ (brain to blood)	*λ*_E2, brn_	1	Teeguarden and Barton [36]

Partition coefficient of E_2_ (gonad to blood)	*λ*_E2, gon_	1	Plowchalk and Teeguarden [60]

Partition coefficient of E_2_ (liver to blood)	*λ*_E2, liv_	3	Watanabe et al. [20]

Partition coefficient of E_2_ ("other" to blood)	*λ*_E2, oth_	1	Plowchalk and Teeguarden [60]

Partition coefficient of T (brain to blood)	*λ*_T, brn_	1	Barton and Andersen [69]

Partition coefficient of T (gonad to blood)	*λ*_T, gon_	1	Barton and Andersen [69]

Partition coefficient of T (liver to blood)	*λ*_T, liv_	1	Barton and Andersen [69]

Partition coefficient of T ("other" to blood)	*λ*_T, oth_	1	Barton and Andersen [69]

Partition coefficient of TB (brain to blood)	*λ*_*TB*, brn_	1	Barton and Andersen [69]

Partition coefficient of TB (gonad to blood)	*λ*_TB, gon_	1	Barton and Andersen [69]

Partition coefficient of TB (liver to blood)	*λ*_TB, liv_	1	Barton and Andersen [69]

Partition coefficient of TB ("other" to blood)	*λ*_TB, oth_	1	Barton and Andersen [69]

^a^, ^b^, and ^c ^were assigned with measured values in each fish; the default values were used only when measured data were missing.

Of the 26 calibrated model parameters, 17 were sensitive model parameters with little or no information available in the open literature (Table 2). Vague prior distributions were used for these 17 model parameters. For example, we could not find a published value for the dissociation constant of E_2 _binding to ER in FHM brain specifically (*K*_d_E2ER, brn_). Denny et al. [15] reported that the dissociation constant of E_2 _binding in female FHM liver cytosol is 8.6 nmol/L. As a result, we assigned a lognormal distribution with a geometric mean of 8.6 and a geometric standard deviation of three, which corresponds to a coefficient of variation equal to 1.5. When no data were available in the open literature, we assigned a uniform or log-uniform prior distribution with a large range bounded by biological plausibility. For example, we know that the EE_2 _partition coefficient for blood to water is around 300 [20], and thus fixed the parameter value at 300. However, there were no published data for the blood to water TB partition coefficient (λ_TB, bld_). Therefore, we assigned a vague prior distribution for λ_TB, bld_, which is a log-uniform distribution with a lower bound of one and an upper bound of 1000.

**Table 2:** Summary statistics for prior and posterior distributions of calibrated model parameters (*n *= 26)

Parameter description	Symbols	Prior Distribution (P1, P2)^a^	Reference	Mean (Posterior Distribution)	Median (Posterior Distribution)	95% Confidence Interval (Posterior Distribution)
Partition coefficient of TB (blood to water)	*λ*_TB, bld_	Loguniform (1, 1.0E+3)	Assumed	7.47	7.47	(5.96, 8.93)

Dissociation constant of E_2 _binding to ER in brain (nmol/L)	*K*_d_E2ER, brn_	Lognormal (8.6, 3)	Denny et al. [15]	1.12	1.08	(0.71, 1.87)

Relative binding affinity of EE_2 _to E_2 _for ER binding	RBA_EE2_E2_	Lognormal (1.66, 3)	Denny et al. [15] Gale et al. [14]	3.24	1.64	(0.030, 16.79)

Relative binding affinity of TB to T for AR binding	RBA_TB_T_	Lognormal (6.03, 3)	Wilson et al. [64]	5.25	4.84	(2.29, 10.76)

Inhibition factor for LH production by bound AR (nmol/L)	*ρ*_d_LH, brn_	LogUniform (0.01, 1.0E+3)	Assumed	0.11	0.10	(0.042, 0.21)

Induction factor for LH production by bound ER (nmol/L)	*ρ*_u___LH, brn_	LogUniform (0.01, 1.0E+3)	Assumed	238	138	(4.23, 864)

Hill coefficient for T production	*n*_T_	Lognormal (1.8, 3)	Murphy et al. [63]	1.03	1.01	(0.93, 1.19)

Proportionality constant relating cholesterol to StAR	*ρ*_Chol, go*n*_	Loguniform (1, 5.0E+3)	Artemenk et al. [70]	2.37	1.83	(1.04, 6.69)

Scaling coefficient of Vmax for E_2 _production in gonad (nmol/hr/mg micro-protein)	sc_Vmax_aro, gon_	Loguniform (2.3E-5, 0.23)	Zhao et al. [71]	1.56E-3	1.53E-3	(1.15E-3, 2.12E-3)

Induction factor of E_2 _production by bound LH (L/nmol)	*ρ*_E2_LHLR, gon_	Loguniform (0.1, 100)	assumed	79.84	82.79	(42.61, 99.15)

Scaling coefficient of Vmax for Vtg production in liver (= Vmax/BodyWeight^0.75^) (nmol/hr/kg^0.75^)	sc_Vmax_Vtg, liv_	Loguniform (1, 1.0E+4)	Watanabe et al. [20]	175	169	(121, 271)

Hill coefficient of Vtg production in liver	*n*_VTG_	Uniform (1, 10)	Assumed	2.88	2.87	(1.97, 3.87)

ER background production rate in liver (nmol/L/hr)	Pbg_ER, liv_	Loguniform (5.0E-5, 0.5)	assumed	0.12	0.12	(0.084, 0.17)

Induction rate constant for ER production in liver (1/hr)	k_ER, liv_	Lognormal (0.08, 3)	Watanabe et al. [20]	0.027	0.025	(5.73E-3, 0.065)

AR background production rate in brain (nmol/L/hr)	Pbg_AR, brn_	Loguniform (5.0E-5, 0.5)	assumed	0.012	0.012	(9.1E-3, 0.015)

Inhibition factor of AR production by free androgens (nmol/L)	K_AR, brn_	Loguniform (5E-4, 5)	assumed	3.95	4.08	(2.15, 4.95)

Magnitude of LH production (nmol/hr)	Mag_LH_	Loguniform (2.7E-7, 2.7E-3)	Schulz et al. [72]	8.86E-6	8.75E-6	(6.29E-6, 1.20E-5)

Error variance of plasma E_2 _concentration in natural log space for unexposed female FHMs	Var_Ln_CE2tot_pla_ngml	Inverse Gamma (2, 1.19)	Bois et al. [73]	0.52	0.51	(0.38, 0.73)

Error variance of plasma T concentration in natural log space for unexposed female FHMs	Var_Ln_CTtot_pla_ngml	Inverse Gamma (2, 0.53)	Bois et al. [73]	0.48	0.47	(0.34, 0.69)

Error variance of plasma VTG concentration in natural log space for unexposed female FHMs	Var_Ln_CVTG_pla_mgml	Inverse Gamma (2, 5.31)	Bois et al. [73]	0.49	0.48	(0.35, 0.68)

Error variance of plasma E_2 _concentration in natural log space for TB-exposed female FHMs	Var_Ln_CE2tot_pla_ngml	Inverse Gamma (2, 1.19)	Bois et al. [73]	0.70	0.69	(0.48, 1.03)

Error variance of plasma T concentration in natural log space for TB-exposed female FHMs	Var_Ln_CTtot_pla_ngml	Inverse Gamma (2, 0.53)	Bois et al. [73]	0.40	0.39	(0.27, 0.60)

Error variance of plasma VTG concentration in natural log space for TB-exposed female FHMs	Var_Ln_CVTG_pla_mgml	Inverse Gamma (2, 5.31)	Bois et al. [73]	5.86	5.72	(3.98, 8.60)

Error variance of plasma E_2 _concentration in natural log space for EE_2_-exposed female FHMs	Var_Ln_CE2tot_pla_ngml	Inverse Gamma (2, 1.19)	Bois et al. [73]	1.43	0.81	(0.22, 6.31)

Error variance of plasma T concentration in natural log space for EE_2_-exposed female FHMs	Var_Ln_CTtot_pla_ngml	Inverse Gamma (2, 0.53)	Bois et al. [73]	0.59	0.34	(0.10, 2.76)

Error variance of plasma VTG concentration in natural log space for EE_2_-exposed female FHMs	Var_Ln_CVTG_pla_mgml	Inverse Gamma (2, 5.31)	Bois et al. [73]	0.73	0.71	(0.51, 1.03)

^a ^Definition of P1 and P2 of prior distributions. Loguniform: P1 = minimum of the sampling range in natural space; P2 = maximum of the sampling range in natural space. Lognormal: P1 = geometric mean (exponential of the mean in log-space); P2 = geometric standard deviation (exponential of the standard deviation in log-space, strictly superior to 1). Uniform: P1 = minimum of the sampling range in natural space; P2 = maximum of the sampling range in natural space. Inverse gamma: P1 = shape; P2 = scale (both of the parameters are strictly positive).

The remaining nine parameters were error variances for plasma E_2_, T, and VTG concentrations in unexposed, TB-exposed, and EE_2_-exposed FHMs. We assumed that the errors followed a lognormal distribution with geometric means equal to the model-predicted concentrations of plasma E_2_, T, and VTG, respectively. The variance was estimated by dividing the experimental data into three different groups: unexposed, TB-exposed, and EE_2_-exposed FHM, respectively; and the error variances of the three reproductive endpoints for each group were estimated [45,47]. For each of the nine error variances, we assigned an Inverse Gamma prior distribution based upon a natural logarithm transformation of the measured plasma E_2_, T and VTG concentrations [20]. An Inverse Gamma prior distribution is the conjugate of a normal distribution [46], which simplifies the model computations.

To perform the Markov Chain Monte Carlo simulations, we used MCSim [48], a software package freely available online http://directory.fsf.org/math/mcsim.html. Four independent Markov chains with random seeds were run for 20,000 iterations. For each of the four chains, we saved the last 10,000 iterations, and extracted one set of model parameters out of every 10. For each calibrated model parameter, convergence was evaluated using the 1,000 iterations from each chain and a potential scale reduction criterion (Rhat) [46]. Acceptable values of Rhat ranged from 1.0 to 1.2; this is essentially a ratio of the calibrated model parameter variance between the four Markov chains to the variance within a chain.

### Model Evaluation

We evaluated the predictive ability of our model by simulating reproductive endpoints (i.e., plasma concentrations of E_2_, T, or VTG) from independent studies. The 1,000 iterations obtained from each Markov chain were pooled, and the 4,000 sets of parameter values were treated as a pool of adult female FHMs. We randomly sampled *n *(number of fish in a study) parameter sets to represent the *n *fish used in the study, and simulated the reproductive endpoints measured for each fish. The detailed simulation procedures followed the methods described by Watanabe et al. [20].

After completing *n *simulations for a study, we predicted each reproductive endpoint based upon our lognormal error model. As described in the Model Calibration section, error variances were estimated during model calibration. Using the model prediction and the estimated variance as two parameters, we randomly sampled from the lognormal distribution for each endpoint in each fish. The sampled values were compared with experimental measurements.

### Prediction of Unmeasured Reproductive Endpoints

To observe EDC effects on unmeasured components of the HPG axis (e.g., ER, AR, and LH), and to observe the effects on reproductive endpoints by a mixture of TB and EE_2_, we did three extra simulations. We simulated liver ER concentration, brain AR concentration, and plasma E_2_, T, VTG, and LH concentrations as a function of time in adult female FHMs exposed to 15 ng TB/L, 10 ng EE_2_/L, or a mixture of 15 ng TB/L and 10 ng EE_2_/L for 48 hours, respectively. The concentrations of TB and EE_2 _were chosen because they are environmentally relevant [12,18,19]. In all three simulations, we used the reported [38] median body weight, GSI, and HSI values in adult female FHMs as input parameters.

## Results and Discussion

### Model Calibration

**Figure 2:**
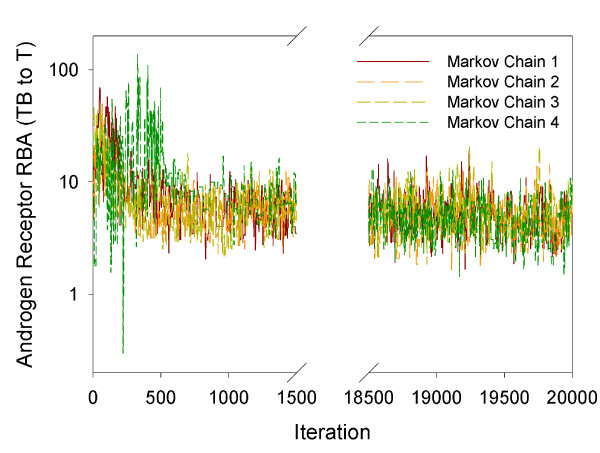
**Four Markov chains**. Androgen receptor relative binding affinity (RBA) for TB relative to T (RBA_TB_T_). This is one of the 26 calibrated model parameters illustrating well-mixed Markov chain trajectories.

A good fit of the experimental data was obtained by running four Markov chains using the Markov Chain Monte Carlo simulations. For the 26 calibrated model parameters, the four Markov chains converged within 20,000 iterations. The model calibration speed is around 12 hours per 100 iterations. The Rhat values of the 26 parameters were all less than 1.2, indicating acceptable convergence. Figure 2 plots the trajectories of the four Markov chains for the relative binding affinity of TB to T (RBA_TB_T_), which is one of the 26 calibrated model parameters. The four chains for this parameter mixed well and converged within 20,000 iterations.

Table 2 includes the summary statistics of posterior distributions for the 26 calibrated parameters. The posterior distribution summary statistics are based on the 4,000 iterations, 1,000 iterations from each of the four chains. In brief, our model improved estimates of 23 model parameters. Of the 26 parameters, 21 had 95% confidence intervals (CIs) narrower than those of their prior distributions; three parameters (i.e., RBA_EE2_E2_, error variances of E_2 _and T for EE_2_-exposed FHMs) had 95% CIs similar to their prior distribution CIs; and two parameters (i.e., error variances of VTG in unexposed and EE_2_-exposed FHMs) had 95% CIs slightly different from their prior distribution CIs. For the error variance of VTG in unexposed FHMs, the upper 95% confidence limit of the posterior distribution was 72% of the 2.5^th ^percentile of its prior distribution. For the error variances of VTG in EE_2_-exposed FHMs, the 95% CIs of the prior and posterior distributions overlapped with each other. But the upper 95% confidence limit of the posterior distribution was only 5% of the 97.5^th ^percentile of its prior distribution. These large differences occurred mainly because the assigned prior distributions for the error variances were based upon experimental data variances, which do not represent the errors exactly, but were good starting points for the model calibration.

It is important to note that the posterior distributions listed in Table 2 are conditional upon fixed model parameters (Table 1), prior distributions of calibrated parameters (Table 2), and the data sets used in calibration. Any change in these components may lead to different posterior distributions of the calibrated parameters. In this study, we carefully searched the literature to assign our model parameters with meaningful and physiologically based values or prior distributions. As additional data become available, our model could be re-calibrated to better define parameter posterior distributions.

### Model Evaluation

In this study, our model was used to simulate experiments ranging in length from 48 hrs to 21 days. The model is capable of simulating longer periods of time, but it does not include a seasonal component. That is, the FHMs simulated in our study were held under laboratory conditions optimal for reproduction and spawn year round. The model could be modified to account for the effect of seasons upon reproduction in order to simulate conditions experienced by wild fish.

### Predictions for Plasma E_2_, T, and VTG Concentrations in Unexposed FHMs

**Figure 3:**
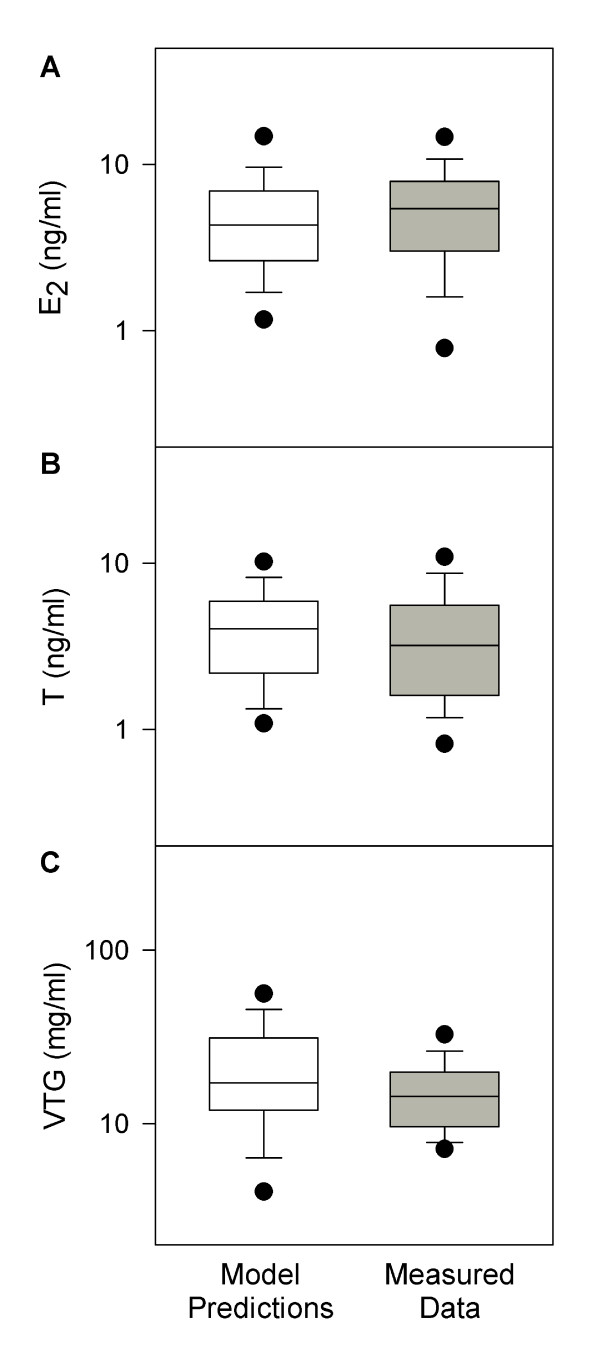
**Comparison of model predictions with measured data in unexposed female FHMs**. *n *= 95. White boxes represent model predictions, and grey boxes represent measured data [38]. The solid line within the box marks the median; the boundary of the box farthest from zero indicates the 75^th ^percentile; the boundary of the box closest to zero indicates the 25^th ^percentile; the whisker (error bar) farthest from zero marks the 90^th ^percentile; whisker (error bar) closest to zero marks the 10^th ^percentile; the circle farthest from zero marks the 95^th ^percentile; and the circle closest to zero marks the 5^th ^percentile.

With the calibrated model parameters, we simulated plasma concentrations of E_2_, T, and VTG in 95 unexposed adult female FHMs [38]. Figure 3 shows a comparison of model predictions and experimental data. For all three endpoints, the mean and median model predictions were within 80 to 150% of the measured means and medians, respectively. Model-predicted 95% CIs encompassed the mean and median measurements, and model-predicted means and medians were within the 95% CIs of the measured data. Thus, in unexposed adult female FHMs, our model successfully predicted all three endpoints (Figure 3). This is an improvement compared to the model for male FHMs [20], which predicted the medians of the measured data, but under-predicted the variances for all three endpoints. Including information from the lognormal error model enabled better predictions of both medians and variances of the measured data.

### Predictions for plasma E2 and VTG concentrations in TB-exposed FHMs

**Figure 4:**
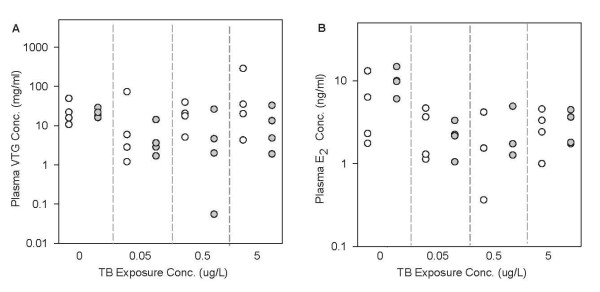
**Comparison of model predictions with measured data in female FHMs exposed to TB for 48 hours**. *n *= 32. White circles represent model predictions, and grey circles represent measured data [39]. Each circle represents one measurement in one fish. (A) plasma VTG concentrations, and (B) plasma E_2 _concentrations. The x-axis represents TB concentrations in μg/L. Note: for panel B, at 0.5 μg TB/L, there are only 3 measured data points.

Figure 4 compares the measured and model-predicted plasma VTG (Figure 4A) and E_2 _(Figure 4B) concentrations in female FHMs exposed to 0, 0.05, 0.5, and 5 μg TB/L for 48 hours [39]. Our model predictions followed the general trend of the measured data, and the model prediction range overlapped with the measured data range for both endpoints at each TB concentration. For plasma VTG concentrations, the median model predictions were within 96% to 579% of the median measurements. The 579% difference seems high, and as a result, we looked into details particularly for this model prediction. We found that this prediction happened when TB concentration equal to 0.5 μg/L. At this TB concentration, we collected plasma VTG concentrations in each of 4 adult female FHMs, which were 4.61, 26.21, 1.99, and 0.06 mg/ml. The last measurement (0.06 mg VTG/ml) is more than 30-fold lower than the second lowest measurement (1.99 mg VTG/ml). As a result, this data point is an outlier, and our model did not capture it. If we exclude this data point, our model predictions (17.63, 20.54, and 5.07 mg/ml) would match the experimental data well. For plasma E_2 _concentrations, the median model predictions were within 44 to 113% of the median measurements. Kolmogorov-Smirnov tests (α = 0.05) showed that the model predictions were not significantly different from the measured data for both plasma VTG and E_2 _concentrations.

**Figure 5:**
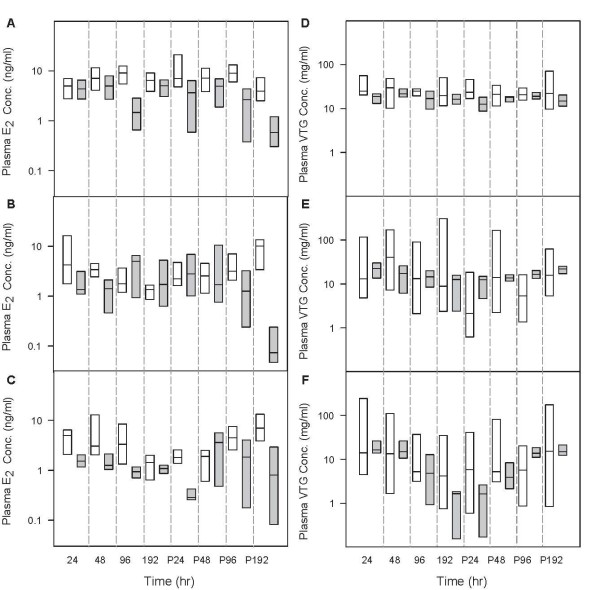
**Comparison of model predictions with measured data in female FHMs exposed to TB for eight days followed by an eight-day depuration**. *n *= 8 at each sampling time. White boxes represent model predictions, and grey boxes represent measured data [40]. The solid line within the box marks the median; the boundary of the box farthest from zero indicates the 75^th ^percentile; the boundary of the box closest to zero indicates the 25^th ^percentile. Because of the small data size (*n *= 8), the plots only show the 50% confidence intervals. (A) plasma E_2 _concentrations in control FHMs, (B) plasma E_2 _concentrations in FHMs exposed to 0.05 μg TB/L, (C) plasma E_2 _concentrations in FHMs exposed to 0.5 μg TB/L, (D) plasma VTG concentrations in control FHMs, (E) plasma VTG concentrations in FHMs exposed to 0.05 μg TB/L, (F) plasma VTG concentrations in FHMs exposed to 0.5 μg TB/L. The x-axis represents time in hours. P24, P48, P96, and P192 represent 24, 48, 96, and 192 hours post-exposure, respectively.

To further evaluate the model's predictive ability for TB-exposed FHMs, we simulated plasma E_2 _and VTG concentrations in FHMs exposed to 0, 0.05, and 0.5 μg TB/L for 8 days followed by an 8-day depuration [40]. For plasma E_2 _concentrations (Figure 5A, B, and 5C), the 95% CIs of model predictions encompassed the medians of the measured data for 16 out of 24 experimental conditions (eight sampling times and three different TB concentrations). Generally, our model predicted the plasma E_2 _concentrations better during the TB exposure phase than during the depuration phase. This is not surprising since we only calibrated the model with experimental data from a TB exposure [7]. In addition, it is interesting to see that the measured plasma E_2 _concentrations declined from the t = P48 to P192 hours for both control FHMs and FHMs exposed to different concentrations of TB. However, the model predictions showed a different trend; that is, for control FHMs, the predicted plasma E_2 _concentrations remained relatively stable throughout the experimental period (Figure 5A); for TB-exposed FHMs, after the exposure, the plasma E_2 _concentrations increased and recovered to concentrations seen in unexposed FHMs. Since the measured plasma E_2 _concentrations decreased in both control FHMs and FHMs exposed to TB, we suspect that there might be some experimental factors that we have not accounted for in the model during the depuration phase.

Figure 5D, E, and 5F compare model-predicted plasma VTG concentrations with the measured data. The median model predictions were within 0.2 to 3.6 fold of the measured median, and the 95% CIs of model predictions encompassed all the measured medians at each sampling time. These results show that the model worked well for predicting the plasma E_2 _and VTG concentrations in female FHMs exposed to 0, 0.05, and 0.5 μg TB/L for eight days.

After being calibrated with the experimental data from Ankley et al. [7], our model accurately predicted the plasma E_2_, T, and VTG concentrations in adult female FHMs exposed to TB. This was achieved by simulating AR-related ligand-receptor binding processes, and by assuming two gene regulation mechanisms: i) down regulation of AR production by free androgens; and ii) down regulation of LH production by bound AR. It is noteworthy that the model was able to accurately fit not only the calibration data (see Additional file 2), but also the VTG and E_2 _data from independent studies by Garcia-Reyero et al. [39] and Ekman et al. [40]. These results indicate that our AR-based modelling framework is plausible, and could be used in studies focused on regulatory aspects of the AR on HPG function. In a recent study, Shoemaker et al. [49] developed a computational model to simulate more detailed biochemical reactions in the FHM steroidogenic pathway. However, their model did not incorporate any AR-related signalling pathways. As AR plays an essential role for androgen responses and subsequent regulation of steroidogenesis, our model advances the work of Shoemaker et al. [49] by simulating AR-related signalling pathways.

The calibration and evaluation results showed that the model was able to predict the three reproductive endpoints from different studies with different experimental conditions. Although the data sets used to calibrate and validate the model were from studies with different experimental designs and analytic methods, the model accounted for the differences and predicted the endpoints well. For example, the calibration data were measured in FHMs exposed to TB for 21 days with a flow-through water exposure design, and the plasma VTG concentrations were measured by a polyclonal FHM-based ELISA [7]. In contrast, one validation data set was from FHMs exposed to TB for 48 hours with a static water exposure design, and plasma VTG concentrations measured using a monoclonal carp-based ELISA [39], while the other validation data set was from FHMs exposed to TB for 8 days followed by an 8 day depuration in a flow-through system, with plasma VTG concentrations measured using the polyclonal FHM-based ELISA. With the parameter set calibrated with the data from one study, our model predicted plasma E_2 _and VTG concentrations comparable to the measurements from the other two studies. This indicates that the model not only fit the data empirically, but also captured major features of the HPG axis in female FHMs exposed to TB. In addition, the two model evaluations also supported the point by Watanabe et al. [20] that the VTG measurements by a polyclonal FHM-based ELISA and by a monoclonal carp-based ELISA are consistent.

### Predictions for plasma VTG concentrations in EE_2_-exposed FHMs

**Figure 6:**
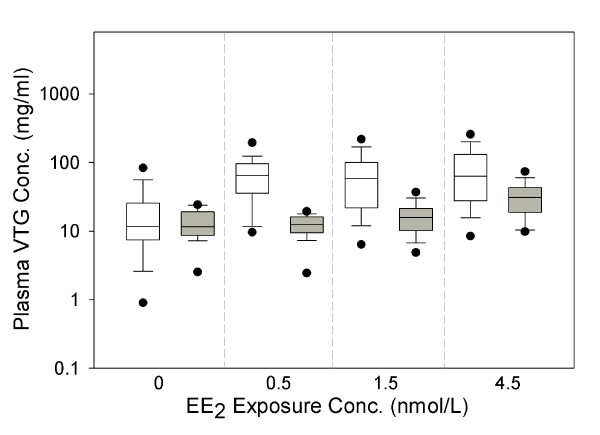
**Comparison of model predictions with measured data in female FHMs exposed to EE_2_**. *n *= 28 at each sampling time. White boxes represent model predictions, and grey boxes represent measured data [42]. The x-axis represents EE_2 _concentrations in ng/L. The solid line within the box marks the median; the boundary of the box farthest from zero indicates the 75^th ^percentile; the boundary of the box closest to zero indicates the 25^th ^percentile; the whisker (error bar) farthest from zero marks the 90^th ^percentile; whisker (error bar) closest to zero marks the 10^th ^percentile; the circle farthest from zero marks the 95^th ^percentile; and the circle closest to zero marks the 5^th ^percentile.

Figure 6 compares model-predicted and measured plasma VTG concentrations in female FHMs exposed to three different concentrations of EE_2 _for 21 days [42]. For the 0.5, 1.5 and 4.5 ng/L exposures, respectively, the 90%, 80%, and 50% CIs of model-predicted VTG concentrations encompassed the medians of the measured data. This trend suggests that the model predicts the endpoint better when the EE_2 _exposure concentration is high and closer to the exposure concentrations used to calibrate the model (i.e., 10 and 100 ng EE_2_/L). For FHMs exposed to 4.5 ng EE_2_/L, the median of our model predictions was around 2 times higher than the measured data, and all measured data were within the 95% CIs of the model predictions. Considering that exposure concentrations less than 10 ng EE_2_/L and exposure durations longer than 8 days are an extrapolation of the model, model predictions of plasma VTG concentrations for the 21-day 4.5 ng EE_2_/L exposure were a surprisingly good fit. The low exposure concentration, longer time frame exposure is more environmentally relevant because EE_2 _concentrations range from 0.5 to 15 ng EE_2_/L in the aquatic environment [50-53], and aquatic animals may be exposed to the chemical throughout their lifetime.

Additionally, we simulated plasma VTG concentrations in FHMs exposed to 1.5 ng EE_2_/L for 21 days as reported by Brian et al. [43]. In total, four control FHMs and four FHMs exposed to EE_2 _were simulated. Brian et al. measured the VTG concentrations with a polyclonal carp VTG ELISA, which uses polyclonal antibodies prepared from carp VTG. In contrast, VTG data used to calibrate the model were measured with a homologous FHM VTG ELISA, which uses polyclonal antibodies prepared from FHM VTG. Direct comparison of the two methods have shown that measurements of FHM plasma VTG concentrations can differ by several orders of magnitude [54]. As a result, instead of comparing the model predictions with the measured data directly, we compared the relative changes of plasma VTG concentrations. The results showed that the range of model-predicted relative change was 0.44 to 4.93, while the range of the measured data relative change was 0.78 to 0.82, all within the range of model predictions.

### Predictions for reproductive endpoints in a mixture of TB and EE_2_

In the next phase of our analysis, we predicted liver ER concentration, brain AR concentration, and plasma E_2_, T, VTG, and luteinizing hormone (LH) concentrations in female FHMs exposed to 15 ng TB/L, 10 ng EE_2_/L, and a mixture of 15 ng TB/L and 10 ng EE_2_/L for 48 hours, respectively. For all endpoints, there was a change after the chemical exposure began followed by a recovery to baseline values after the exposure ended. In panels A, B, and C, after exposure to TB, the plasma E_2_, T, and VTG concentrations followed a trend consistent with the data used in the model calibration and evaluation. After exposure to EE_2_, plasma E_2 _and T concentrations decreased more dramatically than that produced by TB exposure. We did not find any reports of plasma E_2 _or T concentrations in female FHMs exposed to EE_2_. However, in female zebrafish, it was observed that both plasma E_2 _and T concentrations decreased after exposure to 15 ng EE_2_/L for 48 hours [55], which agrees with our model predictions. In addition, plasma VTG concentrations increased after exposure to EE_2_, consistent with the data used to calibrate and evaluate our model. Interestingly, after exposure to a mixture of TB and EE_2_, our model predicted that the plasma E_2 _and T concentrations decreased in an additive manner. In contrast, the plasma VTG concentration increased and followed the trend of an EE_2 _exposure.

**Figure 7:**
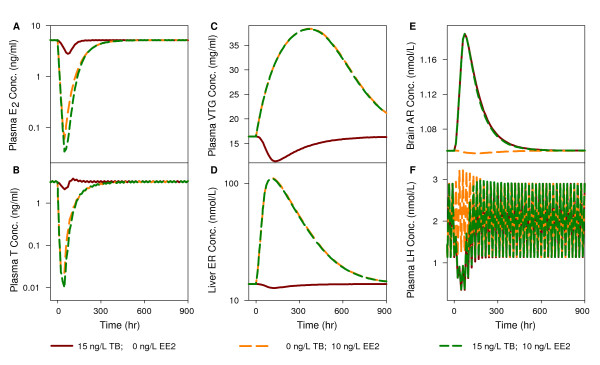
**Model predictions for unmeasured reproductive endpoints in female FHMs**. The predictions are for female FHMs exposed to 15 ng/L TB, 10 ng/L EE_2_, or a mixture of 15 ng/L TB and 10 ng/L EE_2 _for 48 hours, respectively: (A) plasma E_2 _concentration, (B) plasma T concentration, (C) plasma VTG concentration, (D) liver ER concentration, (E) brain AR concentration, and (F) plasma LH concentration.

In panels D, E, and F of Figure 7, we plotted liver ER, brain AR, and plasma LH concentrations, respectively, as a function of time under the three different exposure conditions. Liver ER concentrations were predicted to decrease slightly after exposure to TB, and increase dramatically after exposure to EE_2 _and in response to the mixture. Predicted liver ER concentrations after EE_2 _exposure are consistent with the gene expression data in female FHMs exposed to 10 ng EE_2_/L [56]. Brain AR concentrations were predicted to increase after exposure to TB and the mixture, and decrease slightly after exposure to EE_2_. Plasma LH concentrations were predicted to decrease after exposure to TB and the mixture, and increase slightly after exposure to EE_2 _(consistent with observations in teleosts exposed to EE_2 _[25]). To date, we do not have published data to evaluate model-predicted effects for a mixture of TB and EE_2_. In addition, three of the predicted endpoints (liver ER, brain AR, and plasma LH concentrations) have not been measured in FHM at a protein level because of experimental limitations. However, the predictions can be used to generate hypotheses and help explore possible mechanisms and pathways, which might be tested in the future.

## Conclusions

The model represents the HPG axis in adult female FHM robustly, and predicts plasma E_2_, T and VTG concentrations in female FHMs exposed to TB, EE_2_, or a mixture of TB and EE_2_. This model links environmental estrogen and androgen exposure to changes in apical reproductive endpoints, and serves as a foundation that can be extended to simulate oocyte growth dynamics and other aspects of reproduction. In this study, the model predicted reproductive endpoints from independent studies well. For more than 85% of the simulation results, the 95% CIs of model predictions encompassed the median of the experimental data. To further evaluate the model's predictive ability, more experimental data are needed, especially for the endpoints in FHMs exposed to a mixture of TB and EE_2_.

Important new features of this model include: (i) the simulation of AR in multiple tissue compartments (i.e., brain, liver, and gonad); (ii) AR binding and its effects upon the HPG axis; and (iii) free androgen effects on brain AR concentration. As a result, this model provides a computational framework for endocrine responses of EDCs functioning through both ER and AR.

The model can be used to generate hypotheses to facilitate studies of endocrine responses in female FHMs exposed to other estrogenic EDCs in addition to EE_2_, or other androgenic EDCs in addition to TB. The application can be achieved by defining chemical-specific parameters, such as partition coefficients (e.g., blood to water, or tissue to blood), and binding affinities to ER and AR. Furthermore, the endpoints simulated in this study (i.e. plasma E_2_, T and VTG concentrations) are important determinants affecting egg production in FHMs. In the future, this model could be linked to an oocyte growth dynamics model developed by Li et al. (accepted). Linking these two models would build a connection between EDC effects at a molecular level with effects upon an organism, and thus a population, which is an urgent need in ecological risk assessment.

## Authors' contributions

ZL contributed to model development, simulation, and result analysis. DLV and NDD contributed to model design, and provided experimental data. KJK, KMJ, GTA, JVB, MSS, EFO, JML, MK, and BA provided experimental data. KHW directed the research, and contributed to model development. All authors contributed to, have read, and approved the final version of the manuscript.

## Supplementary Material

Additional file 1 **Differential equations used in the HPG axis model**. The file was created in Microsoft Office Word 2003. The file contains a list of the differential equations used in the HPG axis model for female fathead minnows. Click here for file

Additional file 2 **U-shaped dose-response curves between TB water exposure concentrations and plasma E_2_, T, and VTG concentrations in adult female FHMs**. The file was created in Microsoft Office Word 2003. The file contains three plots for the non-monotonic relationship between TB water exposure concentrations and plasma E_2_, T, and VTG concentrations in adult female FHMs [7]. Click here for file
