# Supplementary material for: A computational model of the hypothalamic - pituitary - gonadal axis in female fathead minnows (Pimephales promelas) exposed to 17α-ethynylestradiol and 17β-trenbolone
Source: BMC Syst Biol. 2011 May 5;5:63. doi: 10.1186/1752-0509-5-63 (PMC3118352; doi:10.1186/1752-0509-5-63)
Supplement: Additional file 1 — Differential equations used in the HPG axis model. The file was created in Microsoft Office Word 2003. The file contains a list of the differential equations used in the HPG axis model for female fathead minnows. [file 1752-0509-5-63-S1.DOC]

# Additional File 1:

# Differential equations used in the HPG axis model

In this file, we include all differential equations used in the HPG model for female FHMs. At the end of each model equation where there is a corresponding equation in the main manuscript, we include the main manuscript equation number in parentheses as a cross reference.

### Brain

$\frac{d(C_{\text{TAR,brn}})}{dt}=k_{\text{1\_TAR,brn}}C_{\text{T,brn}}C_{\text{AR,brn}}-K_{\text{d\_TAR,brn}}k_{\text{1\_TAR,brn}}C_{\text{TAR,brn}}$ (Equation 1)

$\frac{d(C_{\text{E2ER,brn}})}{dt}=k_{\text{1\_E2ER,brn}}C_{\text{E2,brn}}C_{\text{ER,brn}}-K_{\text{d\_E2ER,brn}}k_{\text{1\_E2ER,brn}}C_{\text{E2ER,brn}}$ (Equation 1)

$\frac{d(C_{\text{TBAR,brn}})}{dt}=k_{\text{1\_TBAR,brn}}C_{\text{TB,brn}}C_{\text{AR,brn}}-K_{\text{d\_TBAR,brn}}k_{\text{1\_TBAR,brn}}C_{\text{TBAR,brn}}$ (Equation 1)

$\frac{d(C_{\text{EE2ER,brn}})}{dt}=k_{\text{1\_EE2ER,brn}}C_{\text{EE2,brn}}C_{\text{ER,brn}}-K_{\text{d\_EE2ER,brn}}k_{\text{1\_EE2ER,brn}}C_{\text{EE2ER,brn}}$ (Equation 1)

$\frac{d(C_{\text{LH,brn}}V_{\text{brn}})}{dt}=F_{\text{brn}}(\text{CArt}_{\text{LH}}-\frac{C_{\text{LH,brn}}}{\lambda_{\text{LH,brn}}})+P_{\text{b\_LH,brn}}\times\frac{1+\frac{C_{\text{ER\_bd,brn}}}{\rho_{\text{u\_LH,brn}}}}{1+\frac{C_{\text{AR\_bd,brn}}}{\rho_{\text{d\_LH,brn}}}}$ (Equation 4)

$\begin{matrix} \frac{d(C_{\text{AR\_free,brn}})}{dt}=\frac{\text{Pbg}{}_{\text{AR,brn}}}{1+\frac{(C_{\text{T,brn}}+C_{\text{TB,brn}})}{K_{\text{AR,brn}}}}-(k_{\text{1\_TAR,brn}}C_{\text{T,brn}}C_{\text{AR\_free,brn}}-K_{\text{d\_TAR,brn}}k_{\text{1\_TAR,brn}}C_{\text{TAR,brn}}) \\ -(k_{\text{1\_TBAR,brn}}C_{\text{TB,brn}}C_{\text{AR\_free,brn}}-K_{\text{d\_TBAR,brn}}k_{\text{1\_TBAR,brn}}C_{\text{TBAR,brn}})-k_{\text{e\_AR,}b\text{rn}}C_{\text{AR\_free,brn}} \end{matrix}$ (Equation 5)

$$\begin{matrix} \frac{d(C_{\text{T,brn}}V_{\text{brn}})}{dt}=F_{\text{brn}}(\text{CArt}_{\text{T}}-\frac{C_{\text{T,brn}}}{\lambda_{\text{T,brn}}})-\frac{\text{Vmax}_{\text{aro,brn}}C_{\text{T,brn}}}{\text{Km}_{\text{aro,brn}}+C_{\text{T,brn}}} \\ -V_{\text{brn}}(k_{\text{1\_TAR,brn}}C_{\text{T,brn}}C_{\text{AR,brn}}-K_{\text{d\_TAR,brn}}k_{\text{1\_TAR,brn}}C_{\text{TAR,brn}}) \end{matrix}$$

$$\begin{matrix} \frac{d(C_{\text{E2,brn}}V_{\text{brn}})}{dt}=F_{\text{brn}}(\text{CArt}_{\text{E2}}-\frac{C_{\text{E2,brn}}}{\lambda_{\text{E2,brn}}})+\frac{\text{Vmax}_{\text{aro,brn}}C_{\text{T,brn}}}{\text{Km}_{\text{aro,brn}}+C_{\text{T,brn}}} \\ -V_{\text{brn}} (k_{\text{1\_E2ER,brn}}C_{\text{E2,brn}}C_{\text{ER,brn}}-K_{\text{d\_E2ER,brn}}k_{\text{1\_E2ER,brn}}C_{\text{E2ER,brn}}) \end{matrix}$$

$$\frac{d(C_{\text{Vtg,brn}}V_{\text{brn}})}{dt}=F_{\text{brn}}(\text{CAr}_{\text{Vtg}}-\frac{C_{\text{Vtg,brn}}}{\lambda_{\text{Vtg,brn}}})$$

$$\frac{d(C_{\text{TB,brn}}V_{\text{brn}})}{dt}=F_{\text{brn}}(\text{CArt}_{\text{TB}}-\frac{C_{\text{TB,brn}}}{\lambda_{\text{TB,brn}}})-V_{\text{brn}}(k_{\text{1\_TBAR,brn}}C_{\text{TB,brn}}C_{\text{AR,brn}}-K_{\text{d\_TBAR,brn}}k_{\text{1\_TBAR,brn}}C_{\text{TBAR,brn}})$$

$$\frac{d(C_{\text{EE2,brn}}V_{\text{brn}})}{dt}=F_{\text{brn}}(\text{CArt}_{\text{EE2}}-\frac{C_{\text{EE2,brn}}}{\lambda_{\text{EE2,brn}}}) -V_{\text{brn}} (k_{\text{1\_EE2ER,brn}}C_{\text{EE2,brn}}C_{\text{ER,brn}}-K_{\text{d\_EE2ER,brn}}k_{\text{1\_EE2ER,brn}}C_{\text{EE2ER,brn}})$$

### Gonad

$\frac{d(C_{\text{LHLR,gon}})}{dt}=k_{\text{1\_LHLR,gon}}C_{\text{LH,gon}}C_{\text{LR,gon}}-K_{\text{d\_LHLR,gon}}k_{\text{1\_LHLR,gon}}C_{\text{LHLR,gon}}$ (Equation 1)

$\frac{d(C_{\text{TAR,gon}})}{dt}=k_{\text{1\_TAR,gon}}C_{\text{T,gon}}C_{\text{AR,gon}}-K_{\text{d\_TAR,gon}}k_{\text{1\_TAR,gon}}C_{\text{TAR,gon}}$ (Equation 1)

$\frac{d(C_{\text{E2ER,gon}})}{dt}=k_{\text{1\_E2ER,gon}}C_{\text{E2,gon}}C_{\text{ER,gon}}-K_{\text{d\_E2ER,gon}}k_{\text{1\_E2ER,gon}}C_{\text{E2ER,gon}}$ (Equation 1)

$\frac{d(C_{\text{TBAR,gon}})}{dt}=k_{\text{1\_TBAR,gon}}C_{\text{TB,gon}}C_{\text{AR,gon}}-K_{\text{d\_TBAR,gon}}k_{\text{1\_TBAR,gon}}C_{\text{TBAR,gon}}$ (Equation 1)

$\frac{d(C_{\text{EE2ER,gon}})}{dt}=k_{\text{1\_EE2ER,gon}}C_{\text{EE2,gon}}C_{\text{ER,gon}}-K_{\text{d\_EE2ER,gon}}k_{\text{1\_EE2ER,gon}}C_{\text{EE2ER,gon}}$ (Equation 1)

$$\frac{d(C_{\text{LH,gon}}V_{\text{gon}})}{dt}=F_{\text{gon}}(\text{CArt}_{\text{LH}}-\frac{C_{\text{LH,gon}}}{\lambda_{\text{LH,gon}}})-V_{\text{gon}} (k_{\text{1\_LHLR,gon}}C_{\text{LH,gon}}C_{\text{LR,gon}}-K_{\text{d\_LHLR,gon}}k_{\text{1\_LHLR,gon}}C_{\text{LHLR,gon}})$$

$$\begin{matrix} \frac{d(C_{\text{T,gon}}V_{\text{gon}})}{dt}=F_{\text{gon}}(\text{CArt}_{\text{T}}-\frac{C_{\text{T,gon}}}{\lambda_{\text{T,gon}}})-\frac{k_{\text{E2\_LHLR,gon}}C_{\text{LHLR,gon}}\text{Vmax}_{\text{aro,gon}}C_{\text{T,gon}}}{\text{Km}_{\text{aro,gon}}+C_{\text{T,gon}}} \\ +\frac{\text{Vmax}_{\text{Scc,gon}}C_{\text{Chol,gon}}^{\text{n}_{\text{T}}}}{\text{K}_{\text{0.5Scc,gon}}^{\text{n}_{\text{T}}}+C_{\text{Chol,gon}}^{\text{n}_{\text{T}}}}\div(1+\frac{C_{\text{E2ER,gon}}+C_{\text{EE2ER,gon}}}{K{}_{\text{T}}}) \\ -V_{\text{gon}}(k_{\text{1\_TAR,gon}}C_{\text{T,gon}}C_{\text{AR,gon}}-K_{\text{d\_TAR,gon}}k_{\text{1\_TAR,gon}}C_{\text{TAR,gon}}) \end{matrix}$$

$\frac{d(C_{\text{Vtg,gon}}V_{\text{gon}})}{dt}=F_{\text{gon}}(\text{CArt}_{\text{Vtg}}-\frac{C_{\text{Vtg,gon}}}{\lambda_{\text{Vtg,gon}}})-k_{\text{VTG,gon}}C_{\text{VTG,gon}}V_{\text{gon}}$ (Equation 6)

$\begin{matrix} \frac{d(C_{\text{E2,gon}}V_{\text{gon}})}{dt}=F_{\text{gon}}(\text{CArt}_{\text{E2}}-\frac{C_{\text{E2,gon}}}{\lambda_{\text{E2,gon}}})+\frac{\rho_{\text{E2\_LHLR,gon}}C_{\text{LHLR,gon}}\text{Vmax}_{\text{aro,gon}}C_{\text{T,gon}}}{\text{Km}_{\text{aro,gon}}+C_{\text{T,gon}}} \\ -V_{\text{gon}} (k_{\text{1\_E2ER,gon}}C_{\text{E2,gon}}C_{\text{ER,gon}}-K_{\text{d\_E2ER,gon}}k_{\text{1\_E2ER,gon}}C_{\text{E2ER,gon}}) \end{matrix}$ (Equation 7)

$$\frac{d(C_{\text{TB,gon}}V_{\text{gon}})}{dt}=F_{\text{gon}}(\text{CArt}_{\text{KT}}-\frac{C_{\text{TB,gon}}}{\lambda_{\text{TB,gon}}})-V_{\text{gon}}(k_{\text{1\_TBAR,gon}}C_{\text{TB,gon}}C_{\text{AR,gon}}-K_{\text{d\_TBAR,gon}}k_{\text{1\_TBAR,gon}}C_{\text{TBAR,gon}})$$

$$\frac{d(C_{\text{EE2,gon}}V_{\text{gon}})}{dt}=F_{\text{gon}}(\text{CArt}_{\text{EE2}}-\frac{C_{\text{EE2,gon}}}{\lambda_{\text{EE2,gon}}})-V_{\text{gon}} (k_{\text{1\_EE2ER,gon}}C_{\text{EE2,gon}}C_{\text{ER,gon}}-K_{\text{d\_EE2ER,gon}}k_{\text{1\_EE2ER,gon}}C_{\text{EE2ER,gon}})$$

### Liver

$\begin{matrix} \frac{d(C_{\text{ER,liv}})}{dt}=\text{Pbg}_{\text{ER,liv}}+k_{\text{ER,liv}}(C_{\text{E2ER\_liv}}+C_{\text{EE2ER\_liv}})-(k_{\text{1\_EE2ER,liv}}C_{\text{EE2,liv}}C_{\text{ER,liv}}-K_{\text{d\_EE2ER,liv}}k_{\text{1\_EE2ER,liv}}C_{\text{EE2ER,liv}} \\ -(k_{\text{1\_E2ER,liv}}C_{\text{E2,liv}}C_{\text{ER,liv}}-K_{\text{d\_E2ER,liv}}k_{\text{1\_E2ER,liv}}C_{\text{E2ER,liv}})-k_{\text{e\_ER,liv}}C_{\text{ER,liv}} \end{matrix}$ $\frac{d(C_{\text{TAR,liv}})}{dt}=k_{\text{1\_TAR,liv}}C_{\text{T,liv}}C_{\text{AR,liv}}-K_{\text{d\_TAR,liv}}k_{\text{1\_TAR,liv}}C_{\text{TAR,liv}}$ (Equation 1)

$\frac{d(C_{\text{E2ER,liv}})}{dt}=k_{\text{1\_E2ER,liv}}C_{\text{E2,liv}}C_{\text{ER,liv}}-K_{\text{d\_E2ER,liv}}k_{\text{1\_E2ER,liv}}C_{\text{E2ER,liv}}$ (Equation 1)

$\frac{d(C_{\text{TBAR,liv}})}{dt}=k_{\text{1\_TBAR,liv}}C_{\text{TB,liv}}C_{\text{AR,liv}}-K_{\text{d\_TBAR,liv}}k_{\text{1\_TBAR,liv}}C_{\text{TBAR,liv}}$ (Equation 1)

$\frac{d(C_{\text{EE2ER,liv}})}{dt}=k_{\text{1\_EE2ER,liv}}C_{\text{EE2,liv}}C_{\text{ER,liv}}-K_{\text{d\_EE2ER,liv}}k_{\text{1\_EE2ER,liv}}C_{\text{EE2ER,liv}}$ (Equation 1)

$$\frac{d(C_{\text{LH,liv}}V_{\text{liv}})}{dt}=F_{\text{liv}}(\text{CArt}_{\text{LH}}-\frac{C_{\text{LH,liv}}}{\lambda_{\text{LH,liv}}})$$

$$\frac{d(C_{\text{T,liv}}V_{\text{liv}})}{dt}=F_{\text{liv}}(\text{CArt}_{\text{T}}-\frac{C_{\text{T,liv}}}{\lambda_{\text{T,liv}}})-V_{\text{liv}}(k_{\text{1\_TAR,liv}}C_{\text{T,liv}}C_{\text{AR,liv}}-K_{\text{d\_TAR,liv}}k_{\text{1\_TAR,liv}}C_{\text{TAR,liv}})$$

$$\frac{d(C_{\text{E2,liv}}V_{\text{liv}})}{dt}=F_{\text{liv}}(\text{CArt}_{\text{E2}}-\frac{C_{\text{E2,liv}}}{\lambda_{\text{E2,liv}}})-V_{\text{liv}} (k_{\text{1\_E2ER,liv}}C_{\text{E2,liv}}C_{\text{ER,liv}}-K_{\text{d\_E2ER,liv}}k_{\text{1\_E2ER,liv}}C_{\text{E2ER,liv}})$$

$$\frac{d(C_{\text{Vtg,liv}}V_{\text{liv}})}{dt}=F_{\text{liv}}(\text{CArt}_{\text{Vtg}}-\frac{C_{\text{Vtg,liv}}}{\lambda_{\text{Vtg,liv}}})+\frac{\text{Vmax}_{\text{Vtg,liv}}(C_{\text{E2ER,liv}}+C_{\text{EE2ER,liv}})^{n_{\text{Vtg}}}}{\text{K}_{\text{0.5Vtg,liv}}^{n_{\text{Vtg}}}+(C_{\text{E2ER,liv}}+C_{\text{EE2ER,liv}})^{n_{\text{Vtg}}}}$$

$$\frac{d(C_{\text{TB,liv}}V_{\text{liv}})}{dt}=F_{\text{liv}}(\text{CArt}_{\text{TB}}-\frac{C_{\text{TB,liv}}}{\lambda_{\text{TB,liv}}})-V_{liv}(k_{\text{1\_TBAR,liv}}C_{\text{TB,liv}}C_{\text{AR,liv}}-K_{\text{d\_TBAR,liv}}k_{\text{1\_TBAR,liv}}C_{\text{TBAR,liv}})$$

$$\frac{d(C_{\text{EE2,liv}}V_{\text{liv}})}{dt}=F_{\text{liv}}(\text{CArt}_{\text{EE2}}-\frac{C_{\text{EE2,liv}}}{\lambda_{\text{EE2,liv}}})-V_{\text{liv}} (k_{\text{1\_EE2ER,liv}}C_{\text{EE2,liv}}C_{\text{ER,liv}}-K_{\text{d\_EE2ER,liv}}k_{\text{1\_EE2ER,liv}}C_{\text{EE2ER,liv}})$$

### Other

$$\frac{d(C_{\text{LH,oth}}V_{\text{oth}})}{dt}=F_{\text{oth}}(\text{CArt}_{\text{LH}}-\frac{C_{\text{LH,oth}}}{\lambda_{\text{LH,oth}}})-k_{\text{e\_LH,oth}}C_{\text{LH,oth}}V_{\text{oth}}$$

$$\frac{d(C_{\text{T,oth}}V_{\text{oth}})}{dt}=F_{\text{oth}}(\text{CArt}_{\text{T}}-\frac{C_{\text{T,oth}}}{\lambda_{\text{T,oth}}})-k_{\text{e\_T,oth}}C_{\text{T,oth}}V_{\text{oth}}$$

$$\frac{d(C_{\text{E2,oth}}V_{\text{oth}})}{dt}=F_{\text{oth}}(\text{CArt}_{\text{E2}}-\frac{C_{\text{E2,oth}}}{\lambda_{\text{E2,oth}}})-k_{\text{e\_E2,oth}}C_{\text{E2,oth}}V_{\text{oth}}$$

$$\frac{d(C_{\text{Vtg,oth}}V_{\text{oth}})}{dt}=F_{\text{oth}}(\text{CArt}_{\text{Vtg}}-\frac{C_{\text{Vtg,oth}}}{\lambda_{\text{Vtg,oth}}})-k_{\text{e\_Vtg,oth}}C_{\text{Vtg,oth}}V_{\text{oth}}$$

$\frac{d(C_{\text{TB,oth}}V_{\text{oth}})}{dt}=F_{\text{oth}}(\text{CArt}_{\text{TB}}-\frac{C_{\text{TB,oth}}}{\lambda_{\text{TB,oth}}})-k_{\text{e\_TB,oth}}C_{\text{TB,oth}}V_{\text{oth}}$ (Equation 8)

$$\frac{d(C_{\text{EE2,oth}}V_{\text{oth}})}{dt}=F_{\text{oth}}(\text{CArt}_{\text{EE2}}-\frac{C_{\text{EE2,oth}}}{\lambda_{\text{EE2,oth}}})-k_{\text{e\_EE2,oth}}C_{\text{EE2,oth}}V_{\text{oth}}$$

### Venous blood

$\frac{d(C_{\text{TSBP,ven}})}{dt}=k_{\text{1\_TSBP,ven}}C_{\text{T,ven}}C_{\text{SBP,ven}}-K_{\text{d\_TSBP,ven}}k_{\text{1\_TSBP,ven}}C_{\text{TSBP,ven}}$ (Equation 1)

$\frac{d(C_{\text{E2SBP,ven}})}{dt}=k_{\text{1\_E2SBP,ven}}C_{\text{E2,ven}}C_{\text{SBP,ven}}-K_{\text{d\_E2SBP,ven}}k_{\text{1\_E2SBP,ven}}C_{\text{E2SBP,ven}}$ (Equation 1)

$\frac{d(C_{\text{EE2SBP,ven}})}{dt}=k_{\text{1\_EE2SBP,ven}}C_{\text{EE2,ven}}C_{\text{SBP,ven}}-K_{\text{d\_EE2SBP,ven}}k_{\text{1\_EE2SBP,ven}}C_{\text{EE2SBP,ven}}$ (Equation 1)

$$\begin{matrix} \frac{d(C_{\text{T,ven}}V_{\text{ven}})}{dt}=F_{\text{oth}}\frac{C_{\text{T,oth}}}{\lambda_{\text{T,oth}}}+F_{\text{liv}}\frac{C_{\text{T,liv}}}{\lambda_{\text{T,liv}}}+F_{\text{brn}}\frac{C_{\text{T,brn}}}{\lambda_{\text{T,brn}}}+F_{\text{gon}}\frac{C_{\text{T,gon}}}{\lambda_{\text{T,gon}}}-F_{\text{car}}C_{\text{T,ven}} \\ -V_{\text{ven}}(k_{\text{1\_TSBP,ven}}C_{\text{T,ven}}C_{\text{SBP,ven}}-K_{\text{d\_TSBP,ven}}k_{\text{1\_TSBP,ven}}C_{\text{TSBP,ven}}) \end{matrix}$$

$$\begin{matrix} \frac{d(C_{\text{E2,ven}}V_{\text{ven}})}{dt}=F_{\text{oth}}\frac{C_{\text{E2,oth}}}{\lambda_{\text{E2,oth}}}+F_{\text{liv}}\frac{C_{\text{E2,liv}}}{\lambda_{\text{E2,liv}}}+F_{\text{brn}}\frac{C_{\text{E2,brn}}}{\lambda_{\text{E2,brn}}}+F_{\text{gon}}\frac{C_{\text{E2,gon}}}{\lambda_{\text{E2,gon}}}-F_{\text{car}}C_{\text{E2,ven}} \\ -V_{\text{ven}}(k_{\text{1\_E2SBP,ven}}C_{\text{E2,ven}}C_{\text{SBP,ven}}-K_{\text{d\_E2SBP,ven}}k_{\text{1\_E2SBP,ven}}C_{\text{E2SBP,ven}}) \end{matrix}$$

$$\begin{matrix} \frac{d(C_{\text{EE2,ven}}V_{\text{ven}})}{dt}=F_{\text{oth}}\frac{C_{\text{EE2,oth}}}{\lambda_{\text{EE2,oth}}}+F_{\text{liv}}\frac{C_{\text{EE2,liv}}}{\lambda_{\text{EE2,liv}}}+F_{\text{brn}}\frac{C_{\text{EE2,brn}}}{\lambda_{\text{EE2,brn}}}+F_{\text{gon}}\frac{C_{\text{EE2,gon}}}{\lambda_{\text{EE2,gon}}}-F_{\text{car}}C_{\text{EE2,ven}} \\ -V_{\text{ven}}(k_{\text{1\_EE2SBP,ven}}C_{\text{EE2,ven}}C_{\text{SBP,ven}}-K_{\text{d\_EE2SBP,ven}}k_{\text{1\_EE2SBP,ven}}C_{\text{EE2SBP,ven}}) \end{matrix}$$

### 
