# Supplementary material for: A computational model of the hypothalamic - pituitary - gonadal axis in female fathead minnows (Pimephales promelas) exposed to 17α-ethynylestradiol and 17β-trenbolone
Source: BMC Syst Biol. 2011 May 5;5:63. doi: 10.1186/1752-0509-5-63 (PMC3118352; doi:10.1186/1752-0509-5-63)
Supplement: Additional file 2 — U-shaped dose-response curves between TB water exposure concentrations and plasma E2, T, and VTG concentrations in adult female FHMs. The file was created in Microsoft Office Word 2003. The file contains three plots for the non-monotonic relationship between TB water exposure concentrations and plasma E2, T, and VTG concentrations in adult female FHMs [7]. [file 1752-0509-5-63-S2.DOC]

# Additional file 2:

# U-shaped dose-response curves between TB water exposure concentrations and plasma E_2_, T, and VTG concentrations in adult female FHMs

**Figure AF1:** Comparison of model-predicted plasma E_2_ concentrations with experimental data in adult female FHMs exposed to TB for 21 days . Gray bars represent model predictions, black bars represent experimental data, and error bars represent one standard deviation.


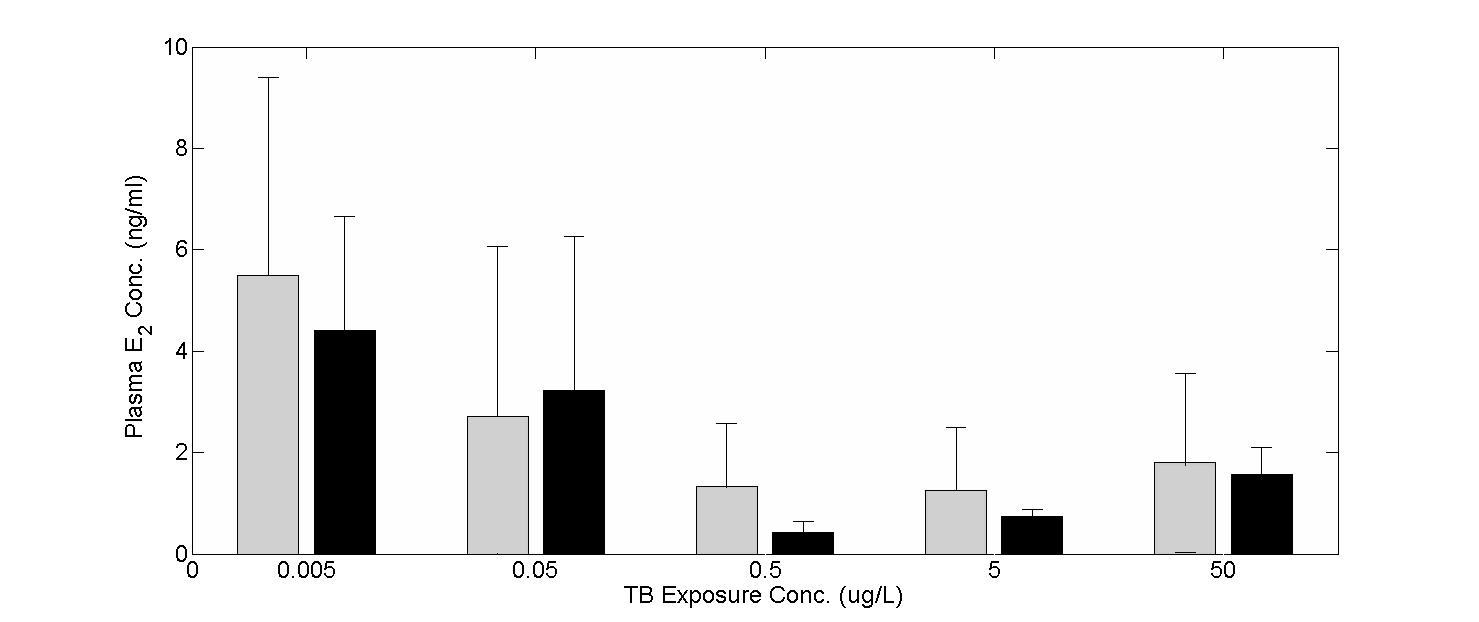


###

**Figure AF2:** Comparison of model-predicted plasma T concentrations with experimental data in adult female FHMs exposed to TB for 21 days . Gray bars represent model predictions, black bars represent experimental data, and error bars represent one standard deviation.


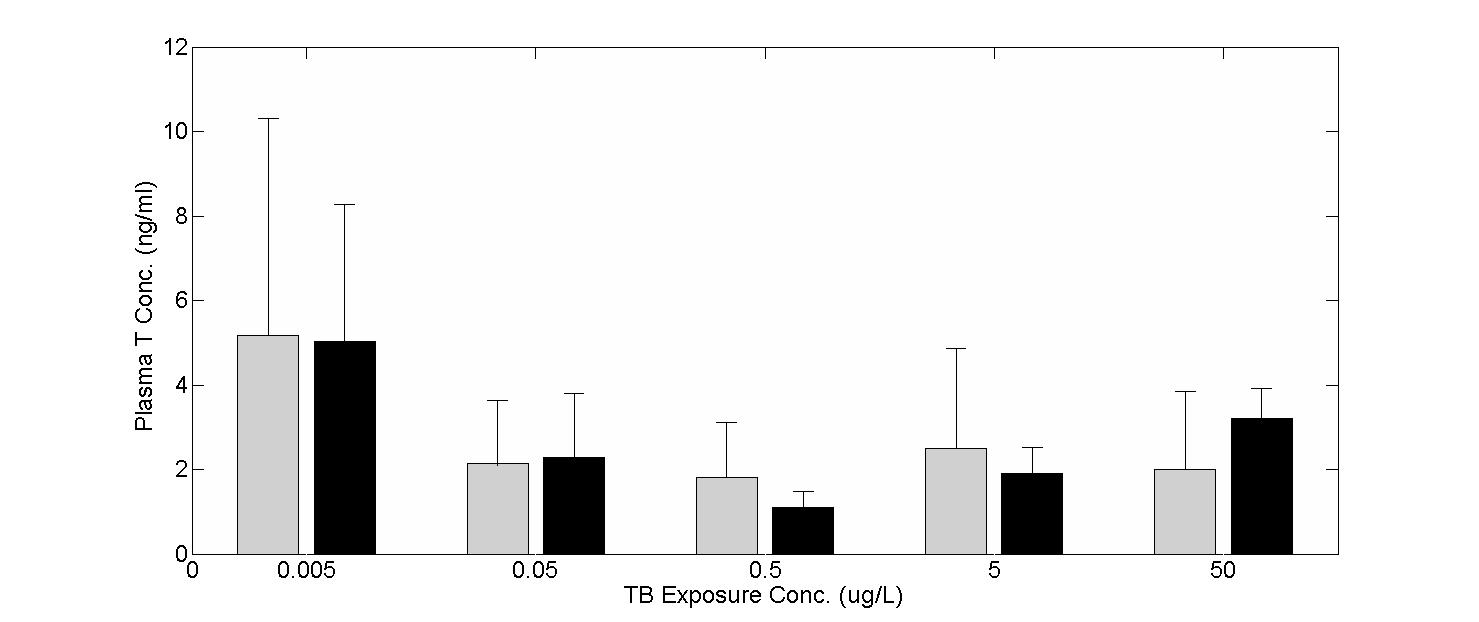


**Figure AF3:** Comparison of model-predicted plasma VTG concentrations with experimental data in adult female FHMs exposed to TB for 21 days . Gray bars represent model predictions, black bars represent experimental data, and error bars represent one standard deviation.


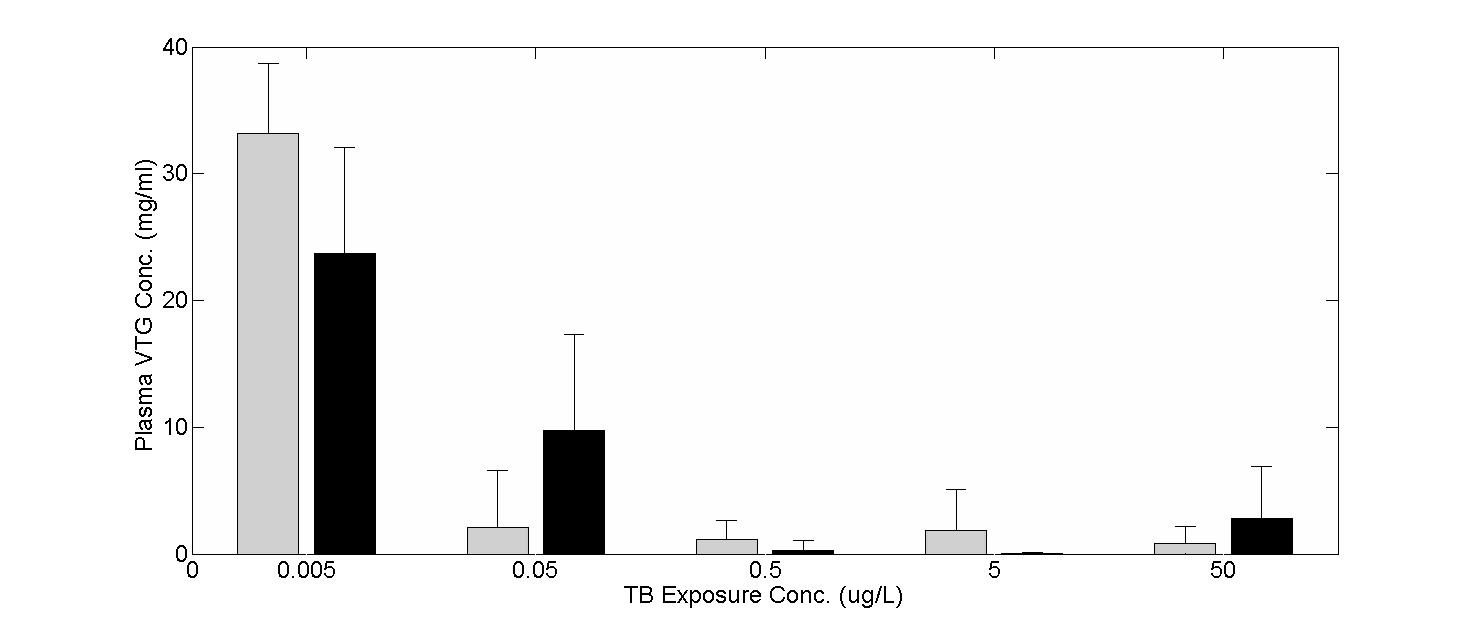


## References cited:

1. Ankley GT, Jensen KM, Makynen EA, Kahl MD, Korte JJ, Hornung MW, Henry TR, Denny JS, Leino RL, Wilson VS, et al: **Effects of the androgenic growth promoter 17β-trenbolone on fecundity and reproductive endocrinology of the fathead minnow.** *Environ Toxicol Chem* 2003, **22:**1350-1360.
